# Parallel analysis of transcription, integration, and sequence of single HIV-1 proviruses

**DOI:** 10.1016/j.cell.2021.12.011

**Published:** 2022-01-20

**Authors:** Kevin B. Einkauf, Matthew R. Osborn, Ce Gao, Weiwei Sun, Xiaoming Sun, Xiaodong Lian, Elizabeth M. Parsons, Gregory T. Gladkov, Kyra W. Seiger, Jane E. Blackmer, Chenyang Jiang, Steven A. Yukl, Eric S. Rosenberg, Xu G. Yu, Mathias Lichterfeld

**Affiliations:** 1 Infectious Disease Division, Brigham and Women’s Hospital, Boston, MA 02115, USA; 2 Ragon Institute of MGH, MIT, and Harvard, Cambridge, MA 02139, USA; 3 San Francisco VA Medical Center, University of California at San Francisco, San Francisco, CA 94121, USA; 4 Infectious Disease Division, Massachusetts General Hospital, Boston, MA 02114, USA; 5 Department of Immunology and Microbiology, Hangzhou Normal University, Zhejiang, P.R. China

**Keywords:** HIV reservoir, proviruses, chromosomal integration site, HIV RNA transcription, antiretroviral treatment, epigenetics

## Abstract

HIV-1-infected cells that persist despite antiretroviral therapy (ART) are frequently considered “transcriptionally silent,” but active viral gene expression may occur in some cells, challenging the concept of viral latency. Applying an assay for profiling the transcriptional activity and the chromosomal locations of individual proviruses, we describe a global genomic and epigenetic map of transcriptionally active and silent proviral species and evaluate their longitudinal evolution in persons receiving suppressive ART. Using genome-wide epigenetic reference data, we show that proviral transcriptional activity is associated with activating epigenetic chromatin features in linear proximity of integration sites and in their inter- and intrachromosomal contact regions. Transcriptionally active proviruses were actively selected against during prolonged ART; however, this pattern was violated by large clones of virally infected cells that may outcompete negative selection forces through elevated intrinsic proliferative activity. Our results suggest that transcriptionally active proviruses are dynamically evolving under selection pressure by host factors.

## Highlights

• A multidimensional assay for HIV-1 reservoir cell profiling is presented (PRIP-seq)
• Transcriptionally active HIV-1 proviruses are actively selected against during ART
• Large transcriptionally active proviral clones resist negative host selection forces
• Epigenetic signals in linear and 3D chromatin contacts influence HIV-1 transcription

## Graphical abstract

**Figure:**
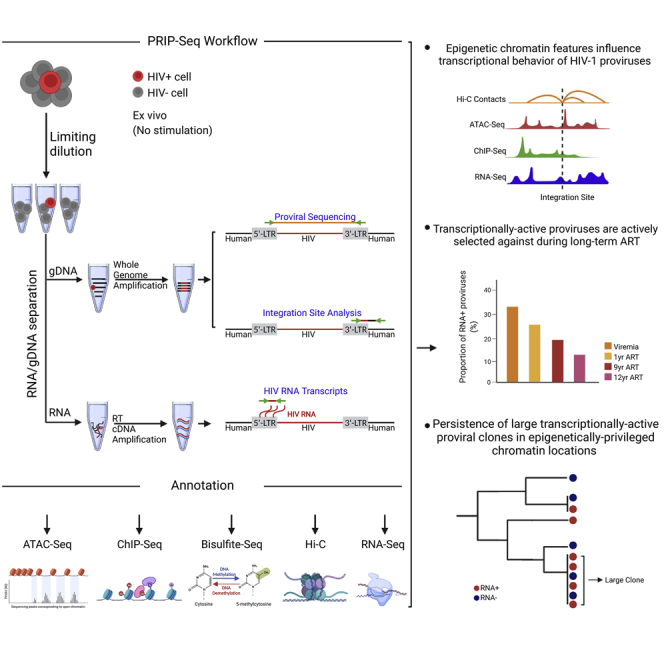

## Teaser

PRIP-seq is a multidimensional single-cell assay that simultaneously captures the proviral sequence, the corresponding chromosomal integration site, and the expression of HIV-1 RNA in single virally infected cells and allows for the global mapping of transcriptionally active and silent proviruses in patients receiving suppressive antiretroviral therapy.

## Introduction

Continuously ongoing, high-level viral replication represents the hallmark of untreated HIV-1 infection. Antiretroviral therapy (ART) inhibits active viral replication, but infected cells harboring chromosomally integrated proviruses persist life long and can drive rebound viremia after treatment interruption, necessitating indefinite ART (Chun et al., 1997; Finzi et al., 1997; Wong et al., 1997). Such residual HIV-1 proviruses have traditionally been regarded as “transcriptionally silent,” but proviral gene expression is not affected by currently available antiretroviral agents and might be ongoing in some cells (Pollack et al., 2017; Wiegand et al., 2017; Yukl et al., 2018). Viral gene expression may represent a key and possibly the only transcriptional feature that distinguishes HIV-1-infected cells from uninfected counterparts; moreover, proviral transcriptional activity can selectively expose HIV-1-infected cells to recognition by adaptive immune responses and increase cell-intrinsic viral cytopathic effects. For these reasons, proviral gene expression might influence the persistence, longevity, and fate of virally infected cells during ongoing suppressive ART.

Footprints of host immune selection mechanisms can be readily detected in untreated people living with HIV-1 (PLHIV) through the emergence of escape mutations in cytotoxic T cell epitopes and in antibody contact regions. However, after initiation of ART, viral mutational escape is effectively blocked, reducing the probability that selection mechanisms can be inferred from viral sequence variations (Antar et al., 2020); instead, selection forces influencing HIV-1-infected cells during suppressive ART may become visible using assays that capture the transcriptional activity and/or the chromosomal location of proviruses. Until recently, HIV-1-infected cells were typically evaluated by PCR assays designed to amplify small segments of HIV-1 DNA, an approach that did not provide information beyond the simple presence or absence of the amplified proviral region (Bruner et al., 2019). Subsequent technical advances permitted the analysis of individual proviral sequences through single-template, near full-length HIV-1 sequencing; in combination with bioinformatic tools designed to discriminate between genome-intact and defective proviruses, this method allowed for the profiling of proviral landscapes at single-genome resolution, frequently revealing clusters of sequence-identical proviral species derived from clonal proliferation of infected cells (Hiener et al., 2017; Ho et al., 2013; Lee et al., 2017; Lorenzi et al., 2016). When preceded by whole-genome amplification, single-genome near full-length proviral sequencing can be performed in conjunction with corresponding chromosomal integration site (IS) analysis (Einkauf et al., 2019; Huang et al., 2021; Patro et al., 2019). This technique, referred to as matched integration site and proviral sequencing (MIP-seq), recently demonstrated a highly disproportionate accumulation of genome-intact proviruses from elite controllers in heterochromatin regions, likely reflecting immune-mediated selection mechanisms that preferentially eliminate proviruses integrated in chromosomal regions more permissive to viral gene expression (Jiang et al., 2020). These studies indirectly supported the assumption that proviral transcriptional activity may increase the vulnerability of HIV-1-infected cells to host immune activity but failed to provide a direct assessment of the transcriptional behavior of individual proviruses. A simultaneous *ex vivo* analysis of the transcriptional activity and chromosomal location of proviruses in participant-derived cells would greatly facilitate the understanding of mechanisms that govern proviral gene expression, influence proviral susceptibility to host immune recognition, and determine immune selection of HIV-1-infected cells during ART.

In this study, we developed an assay for the high-resolution analysis of individual HIV-1-infected cells, designed to simultaneously capture the transcriptional activity, the sequence, and the chromosomal IS of single HIV-1 proviruses. In combination with genome-wide assessments of epigenetic chromatin features from reference data of primary CD4 T cells, this analysis enabled us to measure the frequency of transcriptionally active proviruses, annotate their corresponding chromosomal locations with surrounding epigenetic chromatin features, and profile their longitudinal evolution during continuous ART.

## Results

### Simultaneous analysis of HIV-1 RNA, HIV-1 DNA, and proviral chromosomal integration sites from individual proviral species

**Figure 1:**
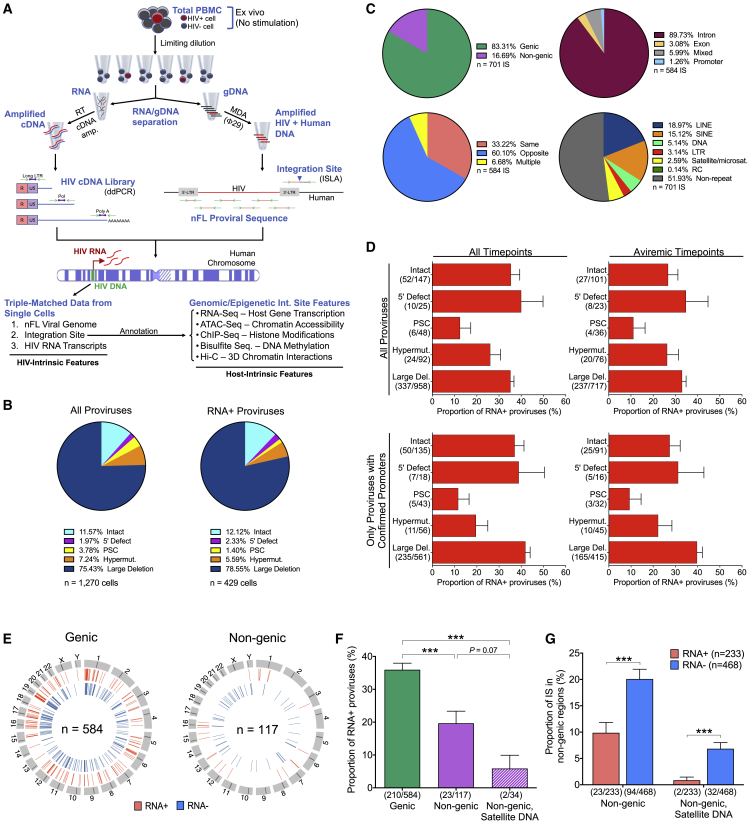
Simultaneous analysis of HIV-1 DNA sequence, integration site, and transcriptional activity from individual infected cells (A) Schematic representation of the PRIP-seq assay design. (B) Proviral sequence classification in all analyzed HIV-1-infected cells and in long LTR RNA-expressing HIV-1-infected cells (PSC, premature stop codon). (C) Proportions of proviruses in genic versus nongenic positions, introns/exons/promoters (genic sites only), same or opposite orientation to host genes (genic sites only), and repetitive genomic elements. (D) Proportion of HIV-1 long LTR RNA-expressing proviruses among analyzed proviruses, stratified according to proviral sequence intactness/defects. Data are shown separately for all proviruses, all proviruses collected during aviremic time points, and for a subset of proviruses with experimentally confirmed intact core promoter regions. (E) Circos plots reflecting the chromosomal locations of transcriptionally active (RNA+) and silent (RNA−) proviruses in genic versus nongenic DNA. (F) Proportion of transcriptionally active proviruses among proviruses integrated in either genic, nongenic, or nongenic satellite DNA regions. (G) Contribution of proviruses in nongenic or nongenic satellite DNA to the total number of transcriptionally active (RNA+) or silent proviruses (RNA−) with detectable chromosomal IS. (E–G) HIV-1 long LTR RNA-expressing proviruses were considered “RNA+.” (^∗∗∗^p < 0.001, Fisher’s exact tests were used for all comparisons. Error bars represent standard errors of proportions ).

**Figure S1:**
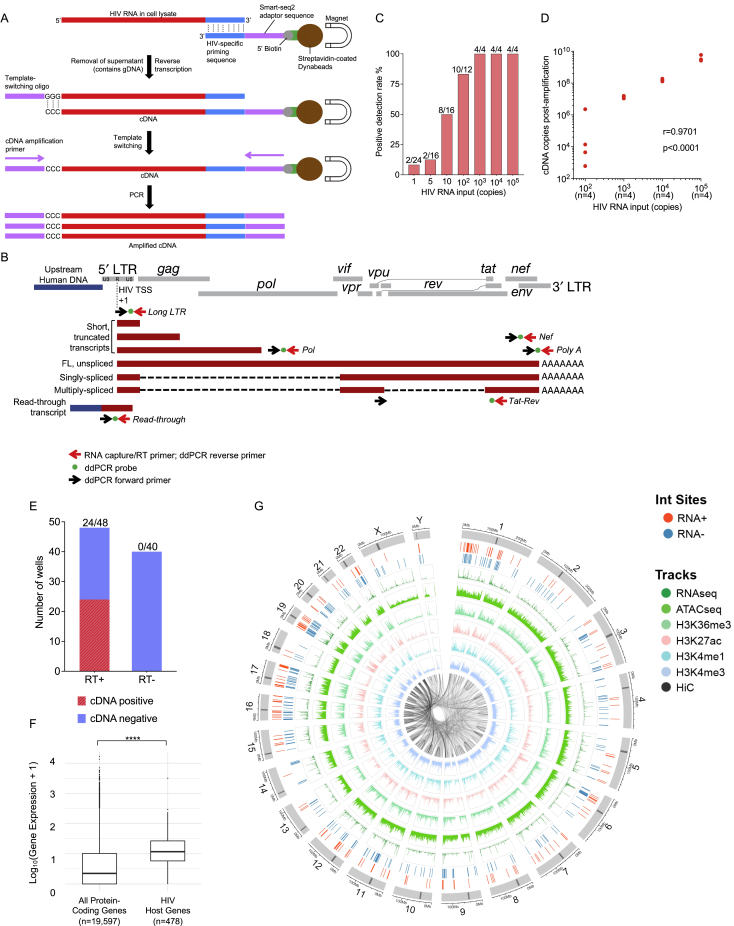
Technical evaluation of PRIP-seq assay, related to Figure 1 (A and B) Schematic representation of the experimental workflow for isolation, reverse transcription, and amplification of HIV-1 RNA/cDNA (A) and of the primer/probe binding sites for ddPCR-based detection of indicated HIV-1 cDNA products (B). (C and D) Known HIV-1 RNA copy numbers were serially diluted in 96-well plates and added to cell lysates of 10,000 PBMC from an HIV-1-uninfected person; afterward, a standard PRIP-seq assay was performed. (C) Proportion of wells with detectable HIV-1 cDNA at the indicated number of input HIV-1 RNA copies. (D) Correlation between input HIV-1 RNA copy numbers and numbers of postamplification HIV-1 cDNA copies detectable by the PRIP-seq assay; Spearman correlation coefficient is shown. (E) Evaluation of possible HIV-1 cDNA contamination by genomic HIV-1 DNA. PRIP-seq was applied to 48 wells, each containing 12,000 PBMC/well from an HIV-infected participant; 40 separate control wells were subjected to the same protocol, except for exclusion of reverse transcriptase from the workflow. Graph demonstrates number of wells with detectable HIV-1 cDNA in samples and controls. (F) Gene expression intensity (determined by RNA-seq) of all human protein-coding genes compared with host genes harboring proviral IS recovered by PRIP-seq in all study subjects. (∗∗∗∗ p < 0.0001, Mann-Whitney U test). (G) Circos plot indicating positioning of long LTR RNA-expressing proviruses (RNA+) and transcriptionally silent (RNA-) proviruses relative to genome-wide assessments of indicated transcriptional (RNA-seq), epigenetic (ATAC-seq and ChIP-seq) and three-dimensional chromatin contact (Hi-C) features. Data from all analyzed proviruses for which IS were available are shown.

We adapted a previously described protocol for simultaneous genome and transcriptome analysis (G&T-seq) (Macaulay et al., 2015) to develop an assay designed for the parallel interrogation of HIV-1 RNA, the HIV-1 chromosomal IS, and the corresponding proviral sequence of individual HIV-1-infected cells, here termed parallel HIV-1 RNA, integration site, and proviral sequencing (PRIP-seq) (Figures 1A, S1A, and S1B). For this purpose, peripheral blood mononuclear cells (PBMC) purified from persons living with HIV-1 were diluted to single HIV-1-infected cells based on Poisson distribution statistics, lysed, and exposed to biotin-labeled primers designed to anneal to HIV-1 RNA. Subsequently, viral RNA transcripts were physically separated using ultrasensitive immunomagnetic enrichment technologies specifically adjusted for the isolation of low-abundance nucleic acids. Viral RNA was then reverse transcribed, followed by cDNA amplification using a Smart-seq2 protocol (Picelli et al., 2014). Next, viral cDNA copies were quantified using droplet digital PCR with primers annealing to the HIV-1 long LTR region and to more elongated HIV-1 RNA transcripts, including those containing HIV-1 pol, nef, tat-rev, and poly-A sequences (Yukl et al., 2018). In parallel, corresponding genomic DNA from each cell lysate was subjected to phi29-catalyzed multiple displacement amplification (MDA), enabling subsequent simultaneous analysis of near full-length proviral sequences and their respective chromosomal IS using a previously described protocol (Einkauf et al., 2019). Briefly, MDA products were split and separately subjected to near full-length HIV-1 amplification as well as amplification of 3′-LTR viral/host junctions by integration site loop amplification (ISLA) assays (Wagner et al., 2014); resulting amplification products were sequenced on the Illumina MiSeq platform. Technical evaluations of this assay platform with defined copy numbers of HIV-1 RNA transcripts spiked into background populations of HIV-1-uninfected cells revealed that as few as 1–5 viral RNA copies could be detected, and there was a 50% probability of detecting 10 HIV-1 RNA copies. Moreover, there was a strong correlation between input HIV-1 RNA copy numbers and the postamplification cDNA copy numbers, indicating that postamplification viral cDNA copies provide a quantitative estimate of the original number of viral RNA molecules present in HIV-1-infected cells (Figures S1C and S1D). Our protocol also reliably excluded contamination of viral RNA with genomic HIV-1 DNA (Figure S1E).

**Figure S2:**
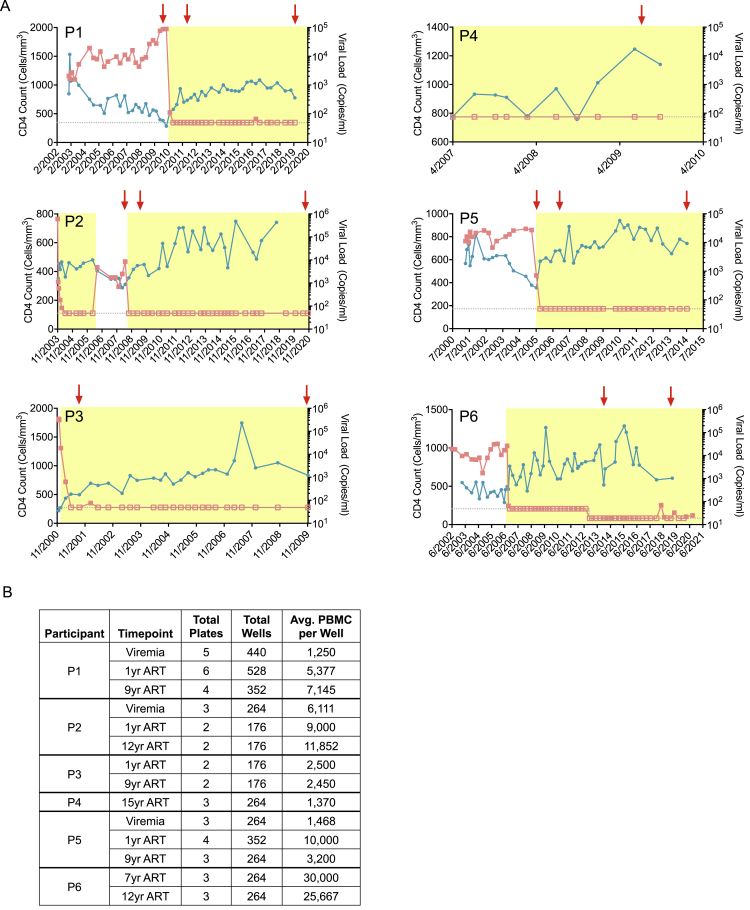
Clinical characteristics of study participants, related to Figure 1 (A) Diagrams reflecting CD4^+^ T cell counts and HIV-1 plasma viral loads of the six study participants (P1–P6). Sampling time points are indicated by red arrows. ART exposure time is indicated by yellow shading. Horizontal dotted lines indicate limits of detection for viral load assays; empty squares indicate participant viral loads at/below the associated limit of detection. (B) Table summarizing number of cells, wells and plates analyzed by PRIP-seq for each participant at indicated PBMC sampling time points.

We applied this complex experimental workflow to PBMC samples that were longitudinally collected from 5 ART-treated PLHIV (P1–P3, P5, P6); in one study participant (P4), a single cross-sectional PBMC sample collected after long-term ART was analyzed (Figure S2). Collectively, we analyzed a total of 1,270 single HIV-1 proviruses from these study participants, of which 147 encoded genome-intact HIV-1; the remaining proviruses displayed various structural sequence defects precluding active viral replication (Figure 1B; Table S1). The chromosomal IS was successfully identified in a total of 701 of these proviral sequences; failure to determine the proviral chromosomal location in the remaining cells was frequently due to the absence of ISLA primer annealing sites in proviruses with large deletions. IS coordinates were most frequently located in introns of highly expressed genes, consistent with previous results (Battivelli et al., 2018; Maldarelli et al., 2014; Schröder et al., 2002); moreover, among proviruses integrated in genes, opposite orientation relative to the host gene was approximately twice as common as concordant/same orientation (Figures 1C and S1F).

Among all 1,270 analyzed proviruses, we noted that HIV-1 RNA transcription varied profoundly: HIV-1 long LTR transcripts were detected in 429 (33.8%) of all 1,270 analyzed HIV-1 proviruses when all sequences were considered, and in 296 (31.1%) of 953 analyzed proviruses when sequences from aviremic time points were selectively evaluated (Figure 1D). Because structural sequence defects in the viral promoter region may compromise the ability of proviruses to express HIV-1 RNA, we used MDA products to amplify and sequence the viral core promoter region in a subset of HIV-1-infected cells; restricting the analysis to proviruses with confirmed intact promoter regions did not markedly affect the relative proportion of transcriptionally active proviruses (Figure 1D). To explore the transcriptional activity of proviruses in the context of epigenetic and architectural features of human genome organization, we annotated all IS coordinates with genome-wide data derived from assays to characterize host gene expression (RNA-seq), chromatin accessibility (ATAC-seq), and three-dimensional (3D) chromosomal contacts (*in situ* Hi-C). RNA-seq and ATAC-seq data were previously generated from primary total CD4 T cells of three ART-treated PLHIV (Einkauf et al., 2019); corresponding Hi-C data from the same three study participants were generated using an *in situ* Hi-C protocol described previously (Díaz et al., 2018) (Figure S1G). In addition, we used ChIP-seq data corresponding to activating and inhibitory histone modifications from primary total CD4 T cells included in the ROADMAP database (Kundaje et al., 2015); genome-wide cytosine methylation data from the iMethyl database (Komaki et al., 2018) were also evaluated to determine epigenetic cytosine methylation in defined regions upstream of HIV-1 promoters. Collectively, these data permitted a comprehensive investigation of the sequence and transcriptional activity of individual proviruses, combined with an analysis of their corresponding chromosomal locations relative to a diverse range of transcriptional, genomic, and epigenetic host features.

### Reduced transcriptional activity of HIV-1 proviruses in nongenic regions

**Figure S3:**
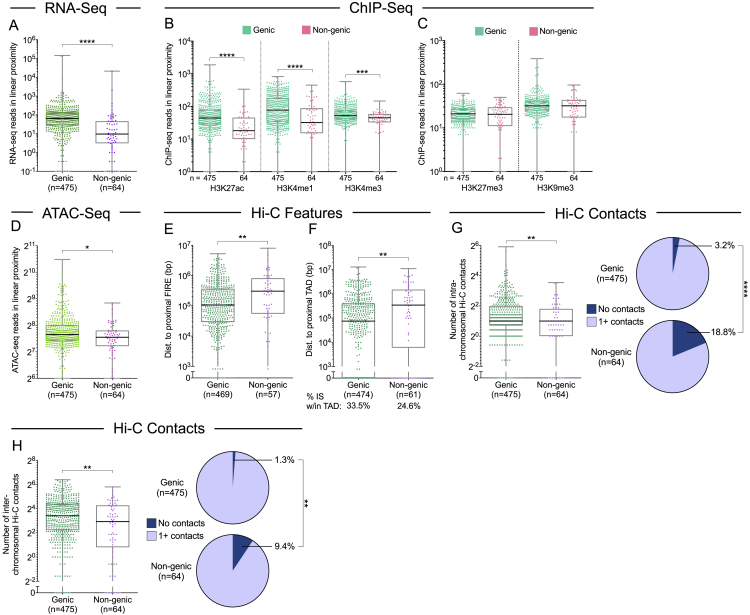
Chromatin features of HIV-1 proviruses integrated in non-genic DNA, related to Figures 1 and 2 (A–D) Sum of local RNA-seq reads (A), ChIP-seq reads corresponding to activating (B), inhibitory (C) histone modifications, and ATAC-seq reads (D) within 5 kb upstream or downstream of proviral IS in genic versus nongenic locations. (E and F) Chromosomal distances of proviruses in genic versus nongenic positions to frequently interacting regions (FIREs) (E) and to topologically associated domains (TADs) (F), determined at 10 kb binning resolution of Hi-C data. (G and H) Numbers of intrachromosomal (G) and interchromosomal (H) contact regions, determined by FiTHiC2-seq (Kaul et al., 2020) (p < 0.05, binning resolution of 20 kb), for proviruses in genic versus nongenic locations. Pie charts reflect proportions of proviruses with no detectable intra- or interchromosomal contacts. (A–H) Clones of proviruses are counted as single datapoints; IS located in chromosomal regions in the ENCODE blacklist (Amemiya et al., 2019) were excluded because of the reduced ability to map next-generation sequencing reads onto repetitive genomic DNA regions. (E and F) Proviral sequences without FIRE annotation by FIREcaller (Crowley et al., 2021) or without TAD annotation by Homer (version 4.10.3) were excluded from the respective analyses. (^∗^p < 0.05, ^∗∗^p < 0.01, ^∗∗∗^p < 0.001, ^∗∗∗∗^p < 0.0001; Mann-Whitney U tests or Fisher’s exact tests were used for all comparisons).

We initially investigated the transcriptional behavior of HIV-1 proviruses located in nongenic regions, which are typically disfavored for proviral integration because of host factors that bias chromosomal insertion sites toward active transcription units in the human genome (Achuthan et al., 2018; Ciuffi et al., 2005). Among all 701 proviruses for which chromosomal IS were identified, 117 (17%) were located in such nongenic/pseudogenic locations (Figure 1E); intact proviruses were more frequently located in nongenic regions relative to defective proviruses (32/122 intact versus 85/579 defective, p = 0.003). Notably, HIV-1 proviruses integrated in intergenic regions of the human genome displayed significantly lower transcriptional activity (Figures 1E–1G); this was particularly true for the small number (n = 34) of HIV-1 proviruses integrated in repetitive satellite or microsatellite DNA, of which the vast majority (94%) were completely transcriptionally silent, consistent with a state of “deep viral latency” (Jordan et al., 2003; Lewinski et al., 2005). Because intergenic DNA is not generally resistant to transcription and RNA complementary to nongenic and satellite DNA is detectable in many species (Smurova and De Wulf, 2018), we explored mechanisms that might explain the repressed transcriptional activity of proviruses integrated in nongenic DNA. As expected, we noted that RNA-seq reads surrounding nongenic locations of integrated HIV-1 DNA were significantly diminished, relative to genic sites harboring integrated HIV-1 DNA (Figure S3A). A similar finding was made for ChIP-seq reads corresponding to activating histone features (H3K27ac, H3K4me1, and H3K4me3); however, no difference was noted for ChIP-seq reads related to inhibitory histone marks (H3K27me3 and H3K9me3) (Figures S3B and S3C). Compared with those in genes, nongenic proviral IS were also in regions with significantly reduced chromatin accessibility, as determined by ATAC-seq (Figure S3D). Interestingly, an alignment of HIV-1 IS to spatial 3D chromatin organization features, evaluated by genome-wide *in situ* Hi-C, demonstrated that nongenic chromosomal regions harboring HIV-1 IS displayed significantly increased chromosomal distances to frequently interacting regions (FIREs) (Schmitt et al., 2016) and to topologically associated domains (TADs) (Beagan and Phillips-Cremins, 2020) (Figures S3E and S3F); moreover, the nongenic regions containing integrated HIV-1 DNA had reduced numbers of 3D intra- and interchromosomal chromatin contacts (Figures S3G–S3H). Together, these results demonstrate that proviruses in nongenic locations have significantly weaker viral transcriptional activity, likely because of nonpermissive genomic and epigenetic chromatin features at chromosomal IS.

### Epigenetic features associated with transcriptional activity of HIV-1 proviruses

**Figure 2:**
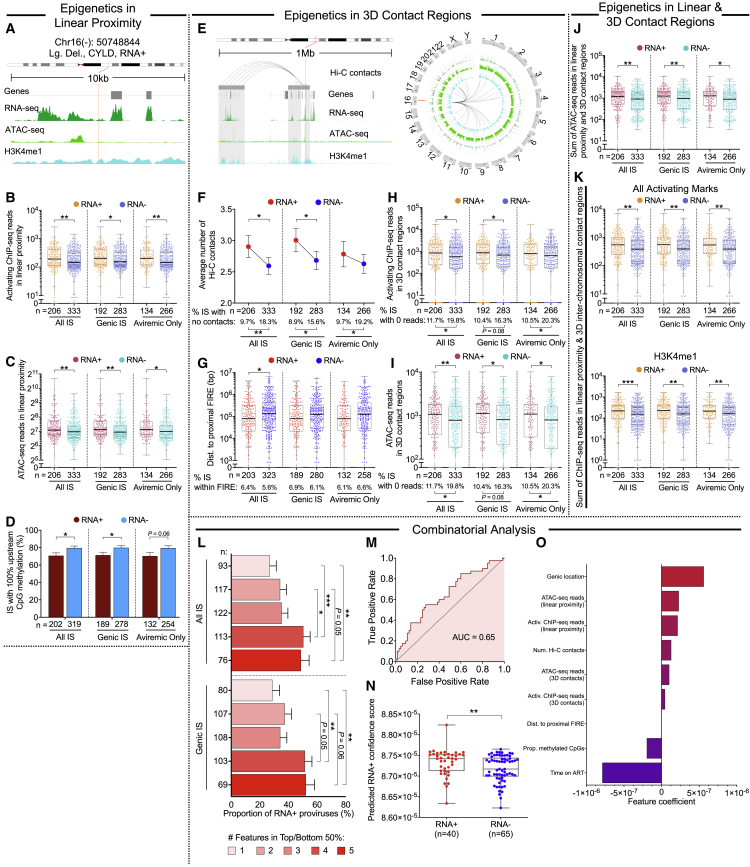
Epigenetic features in linear and three-dimensional contact regions of transcriptionally active proviruses (A) Genome browser snapshot of RNA-seq, ATAC-seq, and ChIP-seq reads in proximity of the indicated representative proviral integration site. (B and C) Dot plots showing ChIP-seq reads corresponding to activating histone features (H3K4me1, H3K4me3, and H3K27ac) (B) and ATAC-seq reads (C) in linear proximity (±5 kb) of RNA-positive or -negative proviruses. (D) Proportion of proviruses with 100% methylated cytosine residues within 2,500 bp upstream of the proviral 5′-LTR HIV-1 promoter. Proviruses with 0 CpGs in this region were excluded. (E) Genome browser snapshot and circos plot highlighting intra- and interchromosomal contact regions of the representative provirus indicated in (A). (F–I) Number of total (intra- and interchromosomal) contacts (F), chromosomal distances to FIREs (G), activating histone-specific ChIP-seq reads in 3D contact regions (H), and ATAC-seq reads in 3D contact regions (I) among HIV-1 RNA-positive or -negative proviruses. In (G), proviral sequences without FIRE annotation by FIREcaller (Crowley et al., 2021) were excluded from the analysis. (J) Sum of ATAC-seq reads in linear (±5 kb) and all 3D contact regions. (K) Sum of activating histone-specific (upper panel) and H3K4me1 (lower panel) ChIP-seq reads in linear (±5 kb) and interchromosomal 3D contact regions. (L) Transcriptional activity of proviruses stratified according to multiple integration site features. Proviruses were categorized based on the number of features within the upper 50^th^ percentile for (B, C, and F) and within the lower 50^th^ percentile for (D and G), relative to the indicated data distributions. (M) Receiver operating characteristic (ROC) curve for a logistic regression model trained to predict proviral transcriptional activity as evaluated on a holdout testing dataset. (N) Dot plot displaying model-predicted confidence scores of HIV-1 RNA expression in RNA-positive or -negative proviruses in the test dataset. (O) Coefficients of each feature in the logistic regression model after training. Positive coefficients are associated with proviral transcriptional activity and negative coefficients are associated with proviral transcriptional silence. (B–D and F–O) HIV-1 long LTR RNA-expressing proviruses were considered “RNA+”; clonal proviral sequences are counted once and shown as RNA+ when at least one member of a clonal cluster had detectable HIV-1 long LTR RNA; IS located in chromosomal regions in the ENCODE blacklist (Amemiya et al., 2019) were excluded. (F–K) Hi-C data at binning resolution of 10 kb are shown. (^∗^p < 0.05, ^∗∗^p < 0.01, ^∗∗∗^p < 0.001, Mann-Whitney U tests or Fisher’s exact tests were used for all comparisons. Error bars in bar diagrams D, F, and L represent SEM or SEP).

**Figure S4:**
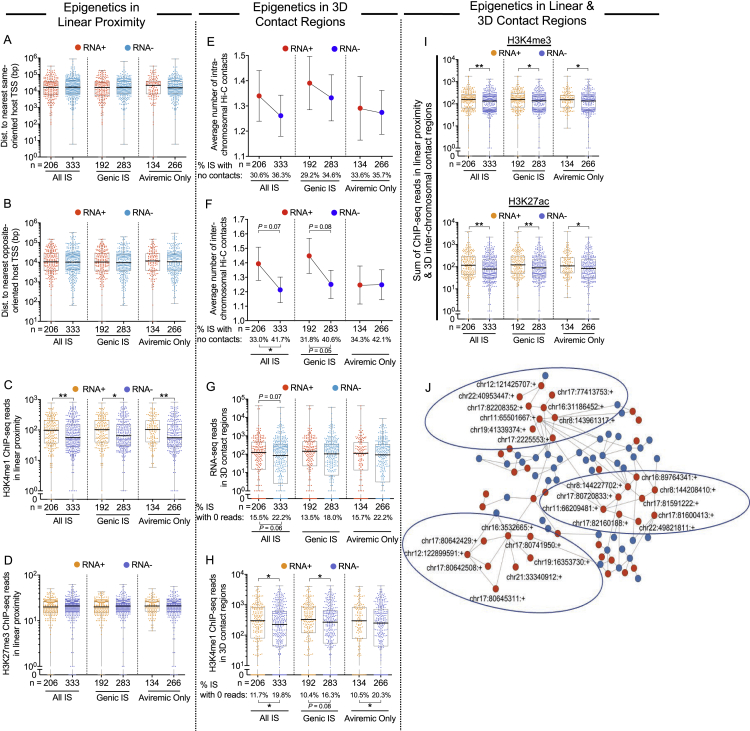
Additional distinguishing features of transcriptionally active HIV-1 proviruses, related to Figure 2 (A and B) Chromosomal distance between transcriptionally active (RNA+) and transcriptionally silent (RNA-) proviruses and the most proximal host transcriptional start site (TSS) in same (A) or opposite (B) orientation. (C and D) H3K4me1- (C) and H3K27me3-specific (D) ChIP-seq reads in linear proximity (±5 kb) to proviral IS. (E and F) Average numbers of intrachromosomal (E) and interchromosomal (F) proviral chromatin contacts; error bars indicate standard error of the mean. (G and H) RNA-seq reads (G) and H3K4me1-specific ChIP-seq reads (H) in all proviral 3D contact regions. (I) Sum of H3K4me3- (upper panel) and H3K27ac-specific (lower panel) ChIP-seq reads in linear proximity and interchromosomal proviral contact regions. (E–I) 3D contacts were determined by Hi-C at 10 kb binning resolution. (J) Network reflecting chromosomal interactions (p < 0.05, 20 kb binning resolution) between IS of transcriptionally active (red) and silent (blue) proviruses from all six study subjects. Circles suggest transcriptional interactomes between HIV-1 RNA+ proviruses. (A–J) HIV-1 long LTR RNA-expressing proviruses were considered “RNA+”; clonal sequences were counted once and were counted as RNA+ when at least one member of a clonal cluster had detectable expression of HIV-1 long LTR RNA. IS located in chromosomal regions in the ENCODE blacklist (Amemiya et al., 2019) were excluded. (^∗^p < 0.05, ^∗∗^p < 0.01, Mann-Whitney U tests or Fisher’s exact tests were used for all comparisons).

To dissect global features associated with the transcriptional activity of proviruses, we analyzed epigenetic characteristics in DNA regions linearly surrounding IS in genic and nongenic regions (Figures 2A–2D and S4A–S4D). Although positioning of IS relative to the most proximal host transcriptional start sites (TSSs) had no noticeable influence on proviral transcription (Figures S4A and S4B), we observed that ATAC-seq reads and ChIP-seq reads corresponding to activating histone modifications (H3K4me1, H3K4me3, and H3K27ac) in linear proximity of HIV-1 DNA IS were significantly higher for transcriptionally active proviruses (Figures 2B, 2C, and S4C); this was true when all proviruses from all time points were considered and when proviruses in genic locations or from aviremic time points were selectively analyzed. Proviral transcriptional silence, on the other hand, was associated with hypermethylated cytosine residues in genomic DNA upstream of the 5′-LTR junction of proviral IS (Figure 2D); inhibitory H3K27me3-specific ChIP-seq marks appeared unrelated to proviral transcriptional behavior (Figure S4D). In a subsequent analysis, we investigated proviral transcriptional activity relative to genomic and epigenetic features in intra- and interchromosomal 3D contact regions, determined by *in situ* Hi-C (Figures 2E–2I). The total number of significant 3D contacts tended to be higher in regions harboring IS of transcriptionally active proviruses, and their chromosomal distances to FIREs were reduced, suggesting that proviral gene expression is facilitated by integration into more interactive chromosomal regions (Figures 2F, 2G, S4E, and S4F). Moreover, there were trends for higher levels of activating histone-specific ChIP-seq reads (Figures 2H and S4H) and ATAC/RNA-seq reads (Figures 2I and S4G) in 3D contact regions of IS of transcriptionally active proviruses. Hypothesizing that combinations of chromatin features in linear, intrachromosomal, and interchromosomal contact regions of chromosomal IS may influence proviral transcriptional behavior, we calculated the sum of reads corresponding to epigenetic features in all three of these chromatin compartments for each individual proviral IS; this composite marker may better approximate the totality of epigenetic signals influencing a given provirus in the 3D spatial configuration of the genome. Collectively, these analyses supported the assumption that combined epigenetic features in linear and 3D contact regions of IS can jointly regulate the transcriptional activity of HIV-1 DNA (Figures 2J, 2K, and S4I). We also observed some evidence for privileged interchromosomal interactions between genomic positions of transcriptionally active proviruses, possibly reflecting the localization of transcriptionally active proviral species at highly permissive and interactive chromatin regions on the outer surface of chromosomal territories (“transcription factories”) (Edelman and Fraser, 2012) (Figure S4J); however, signs of such interchromosomal “transcriptional interactomes” (Schoenfelder et al., 2010) were not observed genome wide, possibly because of the disproportionate overrepresentation of the small, gene-rich chromosomes in 3D interchromosomal interactions (Maass et al., 2019). Collectively, these data suggest that the relative position of proviruses in the nuclear 3D chromatin architecture can influence viral transcription through combined ensemble effects of *cis* and *trans* epigenetic signals and that HIV-1 can take advantage of the full complexity of the human genome and epigenome in its 3D spatial configuration to fine-tune viral transcriptional behavior.

Together, these results identify several genomic and epigenetic features permitting discrimination of transcriptionally active proviruses across a large pool of proviral species isolated from a heterogeneous group of participants and sampling time points; however, the discriminatory effect size of each of these parameters, when assessed as isolated variables, was relatively weak. We subsequently considered a combinatorial analysis of genomic and epigenetic parameters for which significant associations with transcriptional activity were noted in univariable comparisons; these investigations indicated a progressive increase in transcriptional activity among proviruses stratified according to combinations of distinct IS features (Figure 2L). Consistent with this observation, a formal multivariable logistic regression model demonstrated that a combinatorial analysis of genomic and epigenetic IS characteristics facilitated discrimination of transcriptionally active proviruses (Figures 2M–2O) and supported positive associations between activating epigenetic chromatin features and proviral transcriptional activity. However, effects in this model varied considerably with the study subject and were critically influenced by the sampling time point, with longer durations of ART being generally associated with more limited proviral gene expression (Figure 2O). Therefore, the regulation of proviral transcriptional behavior may follow distinct evolutionary pathways in individual subjects, requiring participant-specific longitudinal analyses for further clarification.

### Longitudinal selection of proviral sequences with weaker transcriptional activity

**Figure 3:**
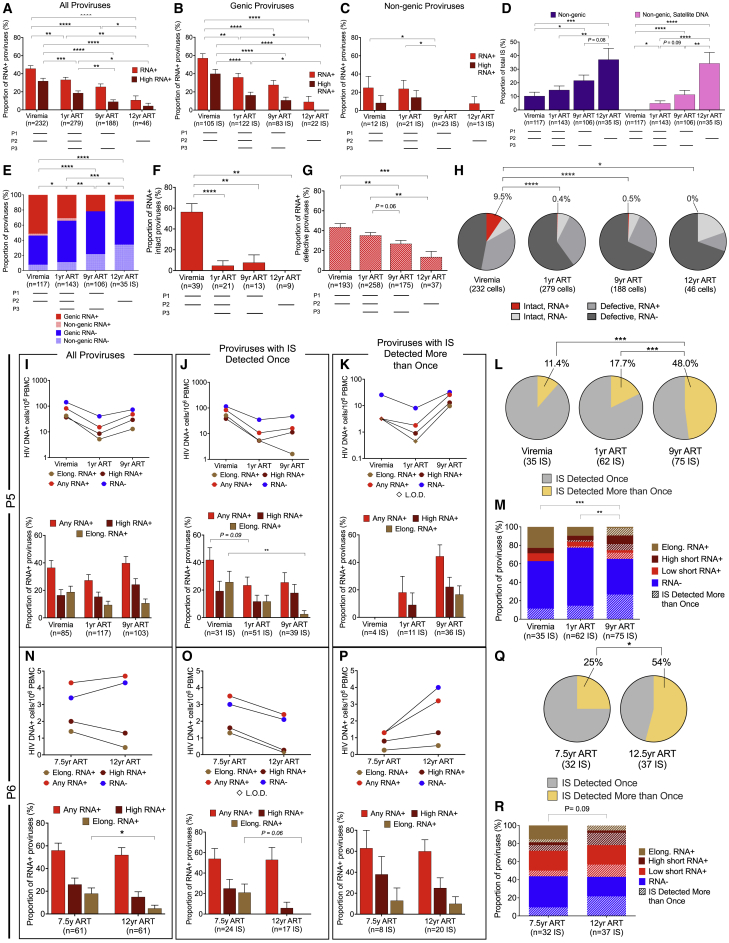
Longitudinal evolution of HIV-1 proviruses (A–C) Relative proportions of proviruses expressing any HIV-1 RNA or high-level (>10,000 postamplification copies) HIV-1 RNA at indicated time points for participants 1–3 (P1–P3). Data for all proviruses (A), proviruses integrated in genic locations (B), and proviruses in nongenic locations (C) are shown; (B) and (C) only include proviruses for which IS are available. (D) Proportion of proviruses integrated in nongenic and nongenic, satellite DNA in a combined longitudinal analysis of participants 1–3. (E) Relative contribution of RNA-positive or -negative proviruses in genic versus nongenic chromosomal locations to the total number of proviruses with known IS in P1–P3. (F and G) Proportions of intact (F) and defective (G) proviruses that were transcriptionally active in P1–P3 at indicated longitudinal time points. (H) Contribution of indicated proviruses to the total number of proviruses in participants 1–3. (A–G) Horizontal dashes indicate available time points from each participant; HIV-1 long LTR RNA-expressing proviruses were considered “RNA+.” (I–K and N–P) Frequencies and proportions of proviruses expressing any HIV-1 RNA, high-level (>10,000 postamplification copies) HIV-1 RNA, elongated HIV-1 RNA (containing pol, nef, spliced tat-rev, or poly-A sequences), or no HIV-1 RNA in study participants 5 (P5, I–K) and 6 (P6, N–P). Data for all proviruses (I and N), for proviruses with IS detected once (J and O), and for proviruses with IS detected more than once (K and P) are shown. (J, K, O, and P) Only include proviruses for which IS are available. (L and Q) Contribution of proviruses with IS detected once or multiple times to the total number of proviruses with known IS in participants 5 (L) and 6 (Q). In (M/R), proviruses are additionally stratified by HIV-1 RNA expression status. (^∗^p < 0.05, ^∗∗^p < 0.01, ^∗∗∗^p < 0.001, ^∗∗∗∗^p < 0.0001, Mann-Whitney U tests, Fisher’s exact tests, or G tests were used for all comparisons. Error bars in bar diagrams represent SEP).

**Figure S5:**
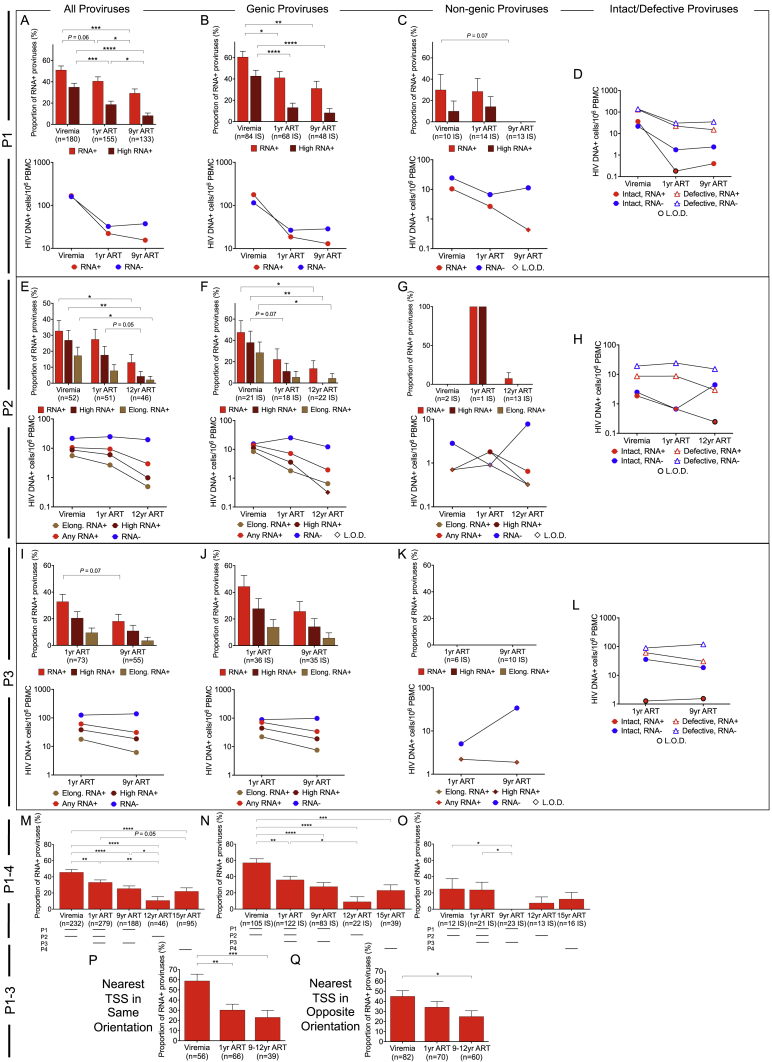
Longitudinal changes in frequency of transcriptionally active and silent proviruses, related to Figure 3 (A–C, E–G, and I–K) Proportions and frequencies of proviruses expressing any HIV-1 RNA, high-level (>10,000 postamplification copies) HIV-1 RNA or elongated HIV-1 RNA (containing pol, nef, spliced tat-rev, or poly-A sequences) at indicated time points in participants 1–3 (P1–P3). Data for all proviruses (A, E, and I), proviruses integrated in genic locations (B, F, and J), and proviruses in nongenic locations (C, G, and K) are shown; (B, C, F, G, J, and K) only include proviruses for which IS are available. (D, H, and L) Frequencies of long LTR RNA-positive or -negative intact or defective proviruses at indicated time points in P1–P3. L.O.D., limit of detection. (M–O) Proportion of long LTR RNA-expressing HIV-1 proviruses in study participants 1–4. Data for all proviruses (M), proviruses in genic locations (N), and proviruses in nongenic locations (O) are shown. Horizontal dashes indicate available time points from each participant. (P and Q) Among proviruses detected once and positioned in either same (P) or opposite (Q) orientation to the nearest host TSS, proportion of proviruses expressing HIV-1 long LTR RNA; longitudinal data are pooled from study subjects 1–3 at indicated time points. (^∗^p < 0.05, ^∗∗^p < 0.01, ^∗∗∗^p < 0.001, ^∗∗∗∗^p < 0.0001, Fisher’s exact tests were used for all comparisons. Error bars in bar diagrams represent SEP).

To investigate the time-dependent evolution of proviruses during suppressive ART, we evaluated the trajectory of HIV-1-infected cells around the time of ART initiation and during the ensuing 9–12 years of therapy in P1 and P2; in P3, data from year 1 and year 9 after ART commencement were available for longitudinal investigation. The frequency of all proviruses tended to decline after ART initiation in these three participants (Figures S5A**–S5L**). However, this decrease was substantially more pronounced for transcriptionally active proviruses, categorized by the detection of any HIV-1 RNA transcript, high-level HIV-1 RNA expression (>10,000 postamplification copies), or elongated HIV-1 RNA transcripts (Figures 3A–3C, S5A, S5E, and S5I). The accumulation of transcriptionally silent proviruses over time coincided with a gradual increase in the frequency of proviral IS in nongenic and satellite DNA (Figure 3D); however, the progressive proportional reduction in HIV-1 RNA-expressing cells was similarly visible among proviruses integrated in genic chromosomal regions (Figures 3B, S5B, S5F, and S5J) and resulted in profound alterations in the composition of the proviral landscape, with a disproportionate overrepresentation of transcriptionally silent proviruses after prolonged ART (Figure 3E). This pattern was consistent with low proportions of transcriptionally active proviruses detected cross-sectionally in study person 4 after 15 years of ART (Figures S5M–S5O). Notably, the longitudinal decline of HIV-1 RNA-expressing proviruses was more obvious for intact HIV-1 DNA compared with defective proviral species, leading to a markedly biased proviral IS profile with almost completely undetectable transcriptionally active intact proviruses after long-term ART (Figures 3F–3H). By contrast, the contribution of transcriptionally silent intact as well as transcriptionally silent defective proviruses to the total pool of HIV-1-infected cells remained relatively stable or expanded over the entire observation period (Figure 3H). Large clones of proviral sequences were detected in two of these study participants after several years of continuous ART. In P2, we noted a large genome-intact clone integrated in the ZNF140 gene and a second clone of near full-length proviruses with a premature stop codon (PSC) in gag integrated in pericentromeric satellite DNA on chromosome 16; both of these proviral clones showed no or minimal transcriptional activity (Figure 5). In P3, we noticed the evolution of two clones of intact proviruses, integrated in centromeric satellite DNA on chromosome 10 and on chromosome Y, respectively; no members of these two clones expressed detectable HIV-1 RNA (Figure 5). Together, these observations suggest a progressive selection of proviruses with lower transcriptional activity and chromosomal integration into nongenic or satellite DNA locations during prolonged ART.

### Persistence of large clones of transcriptionally active proviruses

In contrast to P1–P3, study participant 5 displayed a biphasic evolution of HIV-1-infected cells, with an initial decline occurring during the first year of ART and a subsequent increase during prolonged ART (Figures 3I–3K). A closer investigation demonstrated that this longitudinal pattern was primarily driven by large clones of virally infected cells (Figures 3K–3M) that became visible after prolonged ART durations and frequently expressed viral long LTR HIV-1 RNA and, to a lesser extent, elongated HIV-1 RNA transcripts; in at least two cases, these large, transcriptionally active clones of cells encoded genome-intact HIV-1 (Figure 5). IS of these large, transcriptionally active proviruses were located at chromosomal positions with strong activating epigenetic support (Figure 6). By contrast, among proviruses with IS detected only once (derived from smaller clones or nonclonal HIV-1-infected cells), the proportion of proviruses expressing HIV-1 RNA declined over time (Figure 3J); this longitudinal decrease was most visible in the context of elongated HIV-1 RNA transcripts. A similar pattern was noted in P6, who had detectable low-level plasma viremia (>20 but <100 copies/mL) despite prolonged treatment with ART, in the absence of notable proviral resistance mutations against antiretroviral drugs. In this person, we generally noted a high (>50%) proportion of transcriptionally active proviruses (Figures 3N–3P). Nevertheless, there was a trend for a longitudinal decline of proviruses that were detected once and expressed elongated and/or high-level viral RNA (Figure 3O). By contrast, no such decrease was observed for large proviral clones that remained transcriptionally active and were integrated in chromosomal locations with strong activating chromatin features (Figures 3P–3R, 5, and 6); two of these transcriptionally active clones encoded genome-intact HIV-1 DNA (Figure 5). Collectively, the longitudinal trajectories in P5 and P6 resulted in a proviral landscape characterized by a gradual decline of HIV-1 RNA-expressing proviruses originating from small clones or nonclonal HIV-1-infected cells, coupled with a parallel expansion of large proviral clones frequently displaying strong proviral transcriptional activity. These results are generally consistent with a progressive selection advantage for proviruses with lower transcriptional activity during extended ART. However, large transcriptionally active proviral clones in P5 and P6 appeared to violate this evolutionary pattern and persisted/expanded despite strong expression of viral RNA, possibly because they can outcompete negative host selection forces through enhanced cell proliferation.

### Longitudinal evolution of proviral integration site features

**Figure 4:**
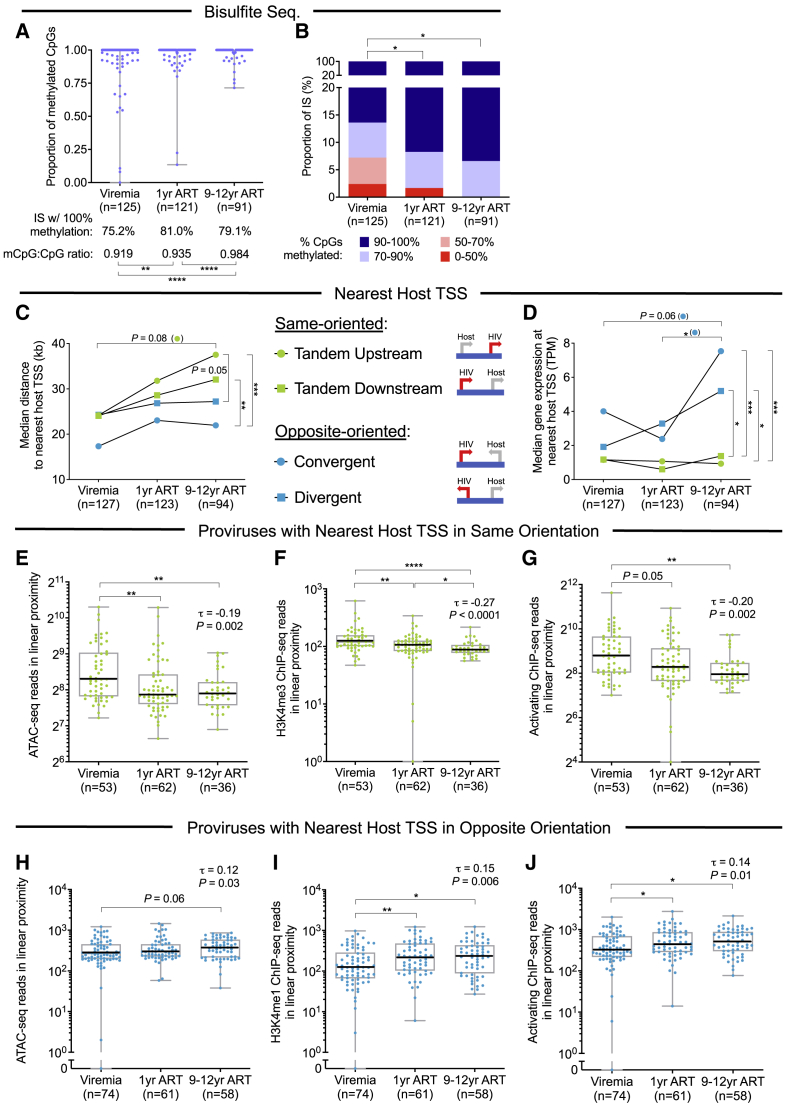
Longitudinal evolution of proviral integration site features (A and B) Proportion of methylated CpG (mCpG) residues within 2,500 bp upstream of the HIV-1 5′-LTR promoter for IS. Proportions of IS with 100% upstream CpG methylation and the average ratio of methylated CpGs to total CpGs are also indicated. Proviruses with 0 CpGs within 2,500 bp upstream of the integration site were excluded. (C) Median distance between proviral IS and the most proximal host transcriptional start site (TSS) with indicated orientation to the proviral sequence. (D) Median RNA-seq-derived gene expression intensity at nearest host TSS with indicated directional orientation to proviral sequence. (E–G) Among proviruses in the same directional orientation as the nearest host TSS, plots indicate the longitudinal evolution of ATAC-seq reads (E) and H3K4me3-specific (F) and all activating (H3K4me1, H3K4me3, and H3K27ac) ChIP-seq reads (G) surrounding (±10 kb) proviral IS. (H–J) Among proviruses in opposite orientation to the nearest host TSS, plots indicate the longitudinal evolution of ATAC-seq reads (H), H3K4me1-specific (I), and all activating (H3K4me1, H3K4me3, and H3K27ac) ChIP-seq reads (J) surrounding (±10 kb) proviral IS. (E–J) Kendall’s rank correlation coefficients (τ) and corresponding p values are indicated in the upper right of each plot. (A–J) Longitudinal data from all proviruses in genic regions from study subjects 1, 2, and 5 are included; IS located in chromosomal regions in the ENCODE blacklist (Amemiya et al., 2019) were excluded; clonal IS are counted only once and assigned to the time point contributing the majority of clonal members or to the earliest time point in the case of a tie. (^∗^p < 0.05, ^∗∗^p < 0.01, ^∗∗∗^p < 0.001, ^∗∗∗∗^p < 0.0001, Mann-Whitney U tests, Fisher’s exact tests, or G tests were used for all comparisons).

To better understand longitudinal selection mechanisms underlying changes in proviral transcriptional activity, we analyzed proviral chromosomal IS features over time in P1, P2, and P5, in whom PRIP-seq data were obtained at relatively similar time points (viremia, 1 year after ART initiation, and 9–12 years after ART initiation). Aligning IS coordinates to genome-wide cytosine methylation data in reference CD4 T cells (Komaki et al., 2018), we noted a progressive longitudinal accumulation of genic proviruses integrated in chromosomal regions with hypermethylated cytosine residues in host DNA upstream of the proviral 5′-LTR promoter, suggesting a role of epigenetic cytosine methylation in silencing proviral transcriptional activity during prolonged ART (Figures 4A and 4B). Although progressive transcriptional silencing was similarly visible for genic proviruses in same and opposite configurations to the most proximal host TSS (Figures S5P and S5Q), we noted marked differences in the longitudinal evolution of IS features for proviruses in the same versus opposite orientations to the nearest host TSS. Although chromosomal distances between proviral IS and the nearest same-oriented TSSs tended to increase over time, this trend was less apparent for chromosomal distances to the nearest opposite-oriented TSSs (Figure 4C). Moreover, using RNA-seq data from primary CD4 T cells from reference datasets (Einkauf et al., 2019), we noted that the host gene expression intensity at the nearest same-oriented TSSs remained stable over time, whereas host transcriptional activity at the nearest opposite-oriented TSSs increased, most notably in the context of TSSs in convergent orientation to the provirus (Figure 4D). In addition, among proviruses with the same orientation as the nearest host TSS, ATAC-seq and activating ChIP-seq reads in linear proximity of regions harboring integrated HIV-1 DNA decreased significantly over time (Figures 4E–4G); by contrast, there was a trend for increasing levels of ATAC-seq and activating ChIP-seq reads in linear proximity of IS for proviruses in opposite orientation to the nearest host TSS (Figures 4H–4J). Together, these data suggest that progressive selection of more transcriptionally silent proviruses over time is partly achieved by two complementary mechanisms: (1) selection forces that promote preferential persistence of proviruses with relatively increased distance to same-oriented host TSSs; transcriptional silencing of these proviruses is likely due to deprived proviral access to activating epigenetic chromatin signals and to host transcriptional machinery; and (2) host factors that select for proviruses in relative proximity to highly expressed host TSSs in opposite orientation; proviral transcriptional repression in this context may be achieved through transcriptional interference from host gene expression (Han et al., 2008; Lenasi et al., 2008).

### Transcriptional activity of clonal HIV-1 proviruses

**Figure 5:**
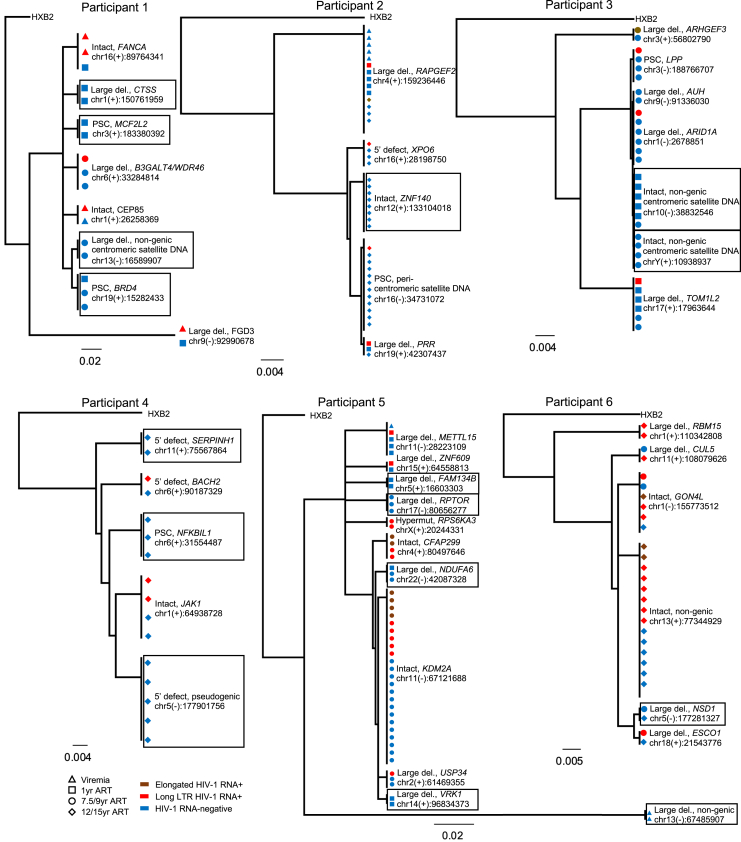
Transcriptional behavior of clonal HIV-1 proviruses Phylogenetic trees of clonal HIV-1 proviruses from the six study participants. Each symbol reflects one single provirus. Proviral sequence calls and host genes harboring IS are indicated. Clones that are transcriptionally silent across all members are boxed. PSC, premature stop codon; large del, large deletion; hypermut, hypermutation.

**Figure 6:**
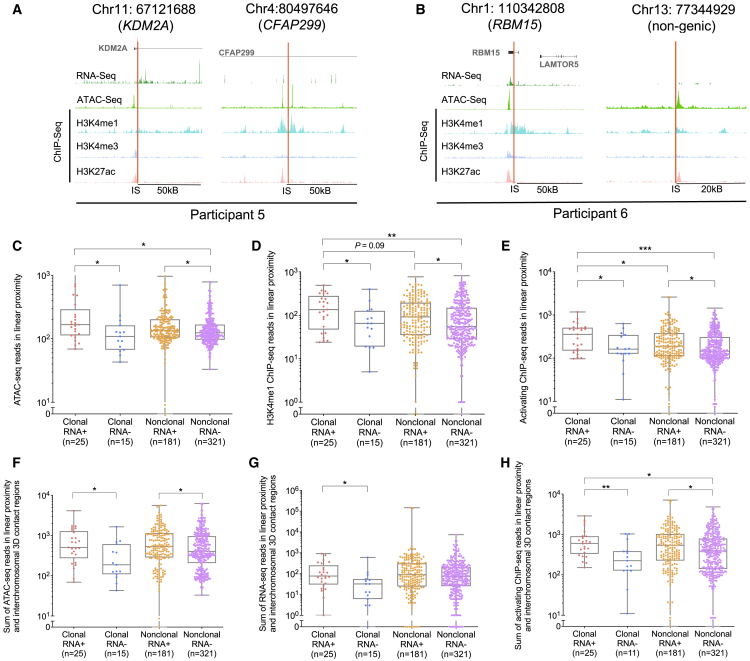
Epigenetic features of transcriptionally active clonal HIV-1 proviruses (A and B) Genome browser snapshots reflecting the local chromatin environment surrounding the proviral IS of selected transcriptionally active clonal proviruses from study persons 5 (A) and 6 (B). (C–E) ATAC-seq (C), H3K4me1-specific ChIP-seq (D), and all activating (H3K4me1, H3K4me3, and H3K27ac) ChIP-seq (E) reads surrounding (±5 kb) the proviral IS of clonal proviruses and of proviruses detected once (here termed “nonclonal”). (F–H) Sum of ATAC-seq (F), RNA-seq (G), and all activating ChIP-seq (H) reads in linear proximity and 3D interchromosomal contact regions of clonal proviruses and proviruses detected once (“nonclonal”) using Hi-C data at 10 kb binning resolution. (C–H) HIV-1 Long LTR RNA-expressing proviruses were considered “RNA+”; HIV-1 Long-LTR RNA-negative proviruses are considered "RNA-". Clonal sequences were counted only once; clones were counted as transcriptionally active when at least one member of a clonal cluster had detectable expression of HIV-1 long LTR RNA. IS located in chromosomal regions in the ENCODE blacklist (Amemiya et al., 2019) were excluded. (^∗^p < 0.05, ^∗∗^p < 0.01, ^∗∗∗^p < 0.001, Mann-Whitney U tests were used for all comparisons).

Clusters of clonal, sequence-identical HIV-1 proviruses were detected in all six study subjects, although their frequencies varied considerably; these sequence clusters (n = 42 in total) result from clonal proliferation of virally infected cells (Bui et al., 2017; Cohn et al., 2015; Hiener et al., 2017; Hosmane et al., 2017; Lee et al., 2017) (Figure 5). Particularly, we noted multiple large clones integrated in nongenic/pseudogenic DNA that evolved after prolonged ART and displayed no or minimal signs of transcriptional activity, specifically in P1–P4. Such clusters of sequence-identical proviruses included a clone with a large deletion integrated in centromeric satellite DNA (P1), a clone with a PSC in pericentromeric satellite DNA (P2), two genome-intact clones in centromeric satellite DNA (P3), and a clone with a 5′-LTR defect in a pseudogenic region (P4). Moreover, one large genome-intact clone integrated in the ZNF140 gene on chromosome 4 was observed after 12 years of treatment in P2; this clone was also completely transcriptionally silent across all 8 member sequences (Figure 5). By contrast, proviral clones with transcriptionally active members in P5 and P6 were frequently integrated in chromosomal locations surrounded by exceptionally strong activating epigenetic chromatin signals in their immediate chromatin proximity, particularly with regard to H3K4me1-specific ChIP-seq reads (Figures 5 and 6A–6E). Similarly, high levels of RNA-seq, ATAC-seq, and activating ChIP-seq signals were observed for transcriptionally active clones when sums of epigenetic sequencing reads in linear proximity and in 3D interchromosomal contact regions of IS were evaluated as composite readouts (Figures 6F–6H). Notably, one large transcriptionally active intact clone was detected in a nongenic region in P6; the IS of this clone was again located in immediate proximity to activating histone modifications, although there were no detectable RNA-seq reads at this nongenic site (Figures 5 and 6B).

The persistence and expansion of large, transcriptionally active proviral clones during long-term ART appeared to violate the longitudinal trajectory of proviral sequences detected once, for which we documented a progressive accumulation of transcriptionally silent proviruses with features of deeper transcriptional latency over time; this raises the question of how transcriptionally active proviruses can resist negative host selection mechanisms. Notably, genes harboring transcriptionally active clonal proviruses were frequently involved in the regulation of cell proliferation and oncogenesis, consistent with previous findings (Maldarelli et al., 2014; Simonetti et al., 2016; Wagner et al., 2014). This was particularly true for a large, transcriptionally active proviral clone integrated into the KDM2A gene (Figures 5 and 6A), which encodes a lysine-specific demethylase that can enhance cell-autonomous proliferation through downregulation of TET2 (Chen et al., 2017b); disruption of TET2 has previously been associated with enhanced clonal proliferation of CAR-encoding T cells (Fraietta et al., 2018). An oncogenic role was also reported for other genes harboring transcriptionally active proviral clones, such as CFAP299/C4orf22 (Kumar et al., 2014), USP34 (Scholtysik et al., 2015), and GON4L (Agarwal et al., 2016) (Figures 5 and 6A). Retroviral integration into such cancer/proliferation-associated genes may result in splicing-induced chimeric host/viral RNA, leading to fusion proteins that can stimulate cell-autonomous clonal proliferation (Cesana et al., 2017; Liu et al., 2020). For other transcriptionally active clonal proviruses, oncogenic functions of genes harboring the proviral IS were less obvious, and proliferation of the corresponding HIV-1-infected cells may be driven by antigen-specific effects (Mendoza et al., 2020; Simonetti et al., 2021). Independently of the mechanisms that drive proliferation of clonal HIV-1-infected cells, we propose that transcriptionally active proviral clones can persist long-term during ART when negative selection forces are outperformed by elevated cell turnover rates that effectively replenish and expand the clonal pool size.

### Responses of individual proviruses to the latency-reversing agents PMA/ionomycin

**Figure 7:**
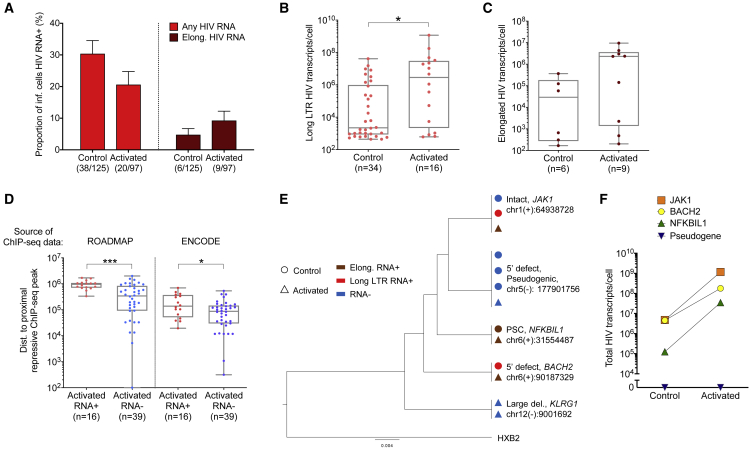
Transcriptional activity of individual proviruses after *in vitro* stimulation (A) Proportion of proviruses producing any HIV-1 RNA or elongated HIV-1 RNA after 12 h of stimulation with PMA/ionomycin or control media. (B and C) Per-cell levels of HIV-1 long LTR (B) and elongated (C) transcripts from single HIV-1-infected cells after 12 h of stimulation with PMA/ionomycin or control media. Only proviruses with detectable HIV-1 RNA are included. (D) Chromosomal distance between proviral IS and nearest ChIP-seq peaks corresponding to repressive histone marks (H3K27me3 and H3K9me3) among viral RNA-positive or -negative proviruses stimulated with PMA/ionomycin. IS are annotated with ChIP-seq data from the ROADMAP project (resting primary CD4^+^ T cells, Kundaje et al., 2015) or from ENCODE (activated primary CD4^+^ T cells ). IS located in chromosomal regions in the ENCODE blacklist (Amemiya et al., 2019) were excluded. (E) Phylogenetic tree of clonal proviral species that were detected in stimulated and nonstimulated experimental conditions. (F) Per-cell levels of total HIV-1 transcripts detected in clonal HIV-1-infected cells analyzed in the presence or absence of stimulation with PMA/ionomycin. (^∗^p < 0.05, ^∗∗∗^p < 0.001, Mann-Whitney U tests were used for all comparisons. Error bars represent SEP).

Latency-reversing agents (LRAs) have been developed for the purpose of enhancing proviral transcriptional activity, with the intention of increasing their susceptibility to host immune factors; however, the influence of such LRAs on transcriptional activity may vary among individual proviruses and has rarely been assessed in single proviruses. To address this, we used the PRIP-seq platform to evaluate the transcriptional activity of HIV-1-infected cells in the presence or absence of stimulation with phorbol 12-myristate 13-acetate (PMA) and ionomycin, activators of protein kinase C (PKC) that are frequently considered the most powerful inducers of HIV-1 gene expression and commonly used as the positive control in HIV-1 reactivation assays. Briefly, participant-derived CD4 T cells were diluted to single HIV-1-infected cells, stimulated with PMA/ionomycin or with control medium for 12 h, and subsequently subjected to PRIP-seq assays. Overall, we assayed a total of 222 proviruses from two different ART-treated participants (P4 and P7); 97 cells were analyzed after stimulation with PMA and 125 cells after treatment with medium alone (Table S2). We observed no significant differences between the proportion of cells producing detectable levels of HIV-1 long LTR or elongated transcripts in stimulated versus nonstimulated cells (Figure 7A); however, among transcriptionally active proviruses, the per-cell levels of any HIV-1 RNA and of elongated HIV-1 RNA were higher after stimulation (Figures 7B and 7C). Remarkably, though, 79.4% of analyzed HIV-1-infected cells failed to produce detectable HIV-1 RNA despite stimulation with PMA/ionomycin; such stimulation-refractory proviruses had IS located in closer proximity to ChIP-seq peaks related to the inhibitory histone modifications H3K9me3 and H3K27me3 (Figure 7D), suggesting that only a subset of proviruses with favorable epigenetic chromosomal IS features can respond to PMA/ionomycin. Notably, for 4 clusters of clonal proviruses, we detected at least one proviral species in both stimulated and unstimulated conditions, allowing for a direct comparison of the transcriptional activity of proviruses with shared IS in the presence or absence of stimulation with PMA/ionomycin (Figures 7E and 7F). In this paired analysis, we observed that proviruses with detectable HIV-1 RNA at baseline uniformly showed higher per-cell levels of viral transcripts after stimulation (Figure 7F). By contrast, a member of a proviral clone with an intact HIV-1 promoter integrated in a pseudogenic position (Chr5: 177901756) that did not produce HIV-1 RNA in the absence of stimulation also remained transcriptionally silent after stimulation (Figure 7F); 5 transcriptionally silent members of this clone were also detected in direct *ex vivo* assessments (P4, Figure 5). Together, these results support the hypothesis that chromosomal location critically determines the susceptibility of proviruses to LRAs; PMA/ionomycin stimulation appears to enhance per-cell levels of viral RNA transcripts but is unable to effectively overcome epigenetic transcriptional blocks and “deep” viral latency in proviruses that are transcriptionally silent at baseline. Future studies will be necessary to determine viral reactivation after stimulation with alternative LRAs.

## Discussion

Although HIV-1 integration in chromosomal DNA is heavily biased toward active host transcription units, the virus can integrate almost anywhere in the human genome, leading to a diverse portfolio of proviral species that differ profoundly with regard to chromosomal location and transcriptional activity (Chen et al., 2017a; Vansant et al., 2020). Seeding such a variegated reservoir of proviral sequences that exist in distinct transcriptional activation states may offer the highest flexibility to resist host immune activity and can be viewed as a probabilistic bet-hedging strategy designed to maximize viral persistence in unpredictable environments (Rouzine et al., 2015). However, how proviral transcriptional behavior and chromosomal location synergize in determining the evolutionary fate and the persistence or elimination of HIV-1-infected cells during ART is unclear. Here, we used an assay to simultaneously profile the transcriptional behavior, the proviral sequence, and the chromosomal position of HIV-1 proviruses in unmanipulated participant-derived cells, permitting the global mapping of transcriptionally active and silent proviruses relative to genome-wide genomic and epigenetic chromatin features and the identification of proviral species that are under evolutionary selection pressure during ART *in vivo*. We note that instead of being “stable” and “transcriptionally silent,” viral reservoir cells are frequently transcriptionally active and dynamically evolving in response to host-dependent selection forces.

Remarkably, our work suggests positive selection of proviruses with lower transcriptional activity during prolonged ART, arguably due to preferential elimination of transcriptionally active proviruses with higher vulnerability to host immune activity. These footprints of host selection strongly suggest that HIV-1-infected cells are subject to host immune surveillance during ART, likely because proviral gene expression can be sensed by host immune factors. In contrast to immune selection effects during untreated HIV-1 infection that can be readily detected by accumulation of viral sequence variations, the selection footprints reported here are indirectly inferred through complex profiling of individual HIV-1-infected cells; identifying the specific immune responses that drive such selection processes will represent a major challenge for future studies. Of note, we repeatedly noticed that large proviral clones can resist evolutionary selection processes that otherwise promote longitudinal accumulation of proviral sequences with progressively decreasing transcriptional activity. Although long-term persistence of transcriptionally active proviruses has also been suggested by other investigators (Halvas et al., 2020; Simonetti et al., 2016), our results suggest that they represent an exception of the rule and are facilitated by two coinciding factors: the presence of strong activating epigenetic signals in linear and 3D contact regions of their IS and an elevated rate of cell turnover that can antagonize negative host selection mechanisms through compensatory repopulation and expansion of the clonal pool size. In addition, upregulation of antiapoptosis and cell survival markers during clonal proliferation of T cells may protect large cell clones against antiviral host immune responses (Cohn et al., 2018; Kuo et al., 2018; Ren et al., 2020). It is possible that these intact, transcriptionally active proviruses play a critical role for driving viral rebound in case of treatment interruptions; future studies will be necessary to address this (Aamer et al., 2020; Cole et al., 2021).

Manipulating the transcriptional behavior of proviruses to enhance their susceptibility to immune-mediated elimination, colloquially referred to as the “shock and kill” approach, has been evaluated in multiple clinical trials (Archin et al., 2012; Rasmussen et al., 2014; Søgaard et al., 2015). Our data suggest that pharmacological increases of proviral transcriptional activity may augment proviral vulnerability to host selection mechanisms, but such effects may have remained invisible in previous clinical trials that primarily used quantitative viral reservoir measurements in bulk CD4 T cells as study endpoints. Indeed, it remains possible that LRAs or immunotherapeutic interventions intensify or accelerate naturally occurring immune selection of proviruses and promote a reservoir structure characterized by features of deeper latency and lower transcriptional activity. These more subtle selection effects may result in a proviral reservoir landscape that is more easily controlled by antiviral host immunity and less likely to fuel rebound viremia; ultimately, such a modulated reservoir structure may allow for a peaceful coexistence between HIV-1 and the host and enable drug-free control of HIV-1 infection (Jiang et al., 2020; Lian et al., 2021). Future experiments on the PRIP-seq platform may help to better dissect the effects of LRAs on single HIV-1-infected cells in samples from clinical trial participants and to detect specific susceptibilities and vulnerabilities of HIV-1 reservoir cells.

### Limitations of the study

A limitation of our work is that transcriptional profiles of individual proviruses were related to chromatin features from reference datasets, and not from single-cell assessments in infected cells. Technical progress in multidimensional single-cell profiling may in the future permit to address this point; in fact, recent studies have started to evaluate lentiviral chromosomal IS coordinates within ATAC-seq reads from single infected cells (Wang et al., 2020). In addition, the transcriptional activity of proviruses is known to be burst driven (Singh et al., 2010; Skupsky et al., 2010), and it is likely that transcriptional noise may have influenced our findings, although our work identifies clear contributions of the genomic environment to the transcriptional regulation of HIV-1 proviruses.

## STAR★Methods

### Key resources table

**Table:** 

REAGENT or RESOURCE	SOURCE	IDENTIFIER
**Antibodies**		

Mouse antihuman CD4 (clone RPA-T4)	BioLegend	Cat#300518; RRID: AB_314086
Mouse antihuman CD3 (clone OKT3)	BioLegend	Cat#317332; RRID: AB_2561943
Mouse antihuman CD45RO (clone UCHL1)	BioLegend	Cat#304236; RRID: AB_2562107
Mouse antihuman CCR7 (clone G043H7)	BioLegend	Cat#353216; RRID: AB_10916386

**Biological samples**		

PBMC samples from study participants living with HIV	Massachusetts General Hospital	https://www.massgeneral.org/

**Chemicals, peptides, and recombinant proteins**		

Buffer RLT Plus	Qiagen	Cat#1053393
Invitrogen Dynabeads MyOne Streptavidin C1	ThermoFisher Scientific	Cat#65002
Invitrogen SUPERase⋅In RNase Inhibitor	ThermoFisher Scientific	Cat#AM2694
Invitrogen dNTP mix (10mM each)	ThermoFisher Scientific	Cat#18427088
AMPure XP beads	Beckman Coulter	Cat#A63882
5M NaCl	ThermoFisher Scientific	Cat#AM9760G
10M NaOH	Millipore Sigma	Cat#72068
0.5M EDTA (pH 8.0)	Promega	Cat#V4231
UltraPure 1M Tris-HCI Buffer (pH 7.5)	ThermoFisher Scientific	Cat#15567027
1M MgCl_2_	ThermoFisher Scientific	Cat#AM9530G
2M KCl	ThermoFisher Scientific	Cat#AM9640G
1M DTT	Millipore Sigma	Cat#646563
TWEEN 20 (50% Solution)	ThermoFisher Scientific	Cat#003005
Betaine solution (5M)	Millipore Sigma	Cat#B0300
BioLegend Cell Activation Cocktail (without Brefeldin A)	BioLegend	Cat#423302
Recombinant IL-2	NIH AIDS Reagent program	www.hivreagentprogram.org
AZT	NIH AIDS Reagent Program	www.hivreagentprogram.org

**Critical commercial assays**		

DNeasy Blood and Tissue Kit	Qiagen	Cat#69504
ddPCR Supermix for Probes (No dUTP)	Bio-Rad	Cat#1863024
Invitrogen SuperScript II Reverse Transcriptase kit	ThermoFisher Scientific	Cat#18064022
KAPA HiFi HotStart ReadyMix	Roche	Cat#7958935001
REPLI-g Single Cell Kit	Qiagen	Cat#150345
REPLI-g Advanced Single Cell Kit	Qiagen	Cat#150365
Invitrogen Platinum Taq DNA Polymerase High Fidelity	ThermoFisher Scientific	Cat#11304102
Stemcell EasySep Human CD4+ T Cell Isolation Kit	Stemcell Technologies	Cat#17952
PicoPure RNA Isolation Kit	Applied Biosystems	Cat#0204

**Deposited data**		

Ensembl (v86)	Ensembl	http://oct2016.archive.ensembl.org/index.html
UCSC Genome Browser	UCSC	http://genome.ucsc.edu
GENCODE (v32)	GENCODE	https://www.gencodegenes.org/human/release_32.html
Roadmap database	Kundaje et al., 2015	http://www.roadmapepigenomics.org/
ENCODE database	(ENCODE Project Consortium, 2012)	https://www.encodeproject.org/
iMethyl database	Komaki et al., 2018	http://imethyl.iwate-megabank.org/
Hi-C data from CD4 T cells	This study	GEO ID: GSE168337
ATAC-Seq and RNA-Seq data from CD4 T cells	Jiang et al., 2020	GEO ID: GSE144334

**Oligonucleotides**		

See Table S3 for List of Primers/Probes	Millipore Sigma/IDT/Qiagen	N/A
Quantitative Synthetic Human immunodeficiency virus 1 (HIV-1) RNA	ATCC	Cat#VR-3245S

**Software and algorithms**		

QuantaSoft software	Bio-Rad	Cat#1864011
Ultracycler v1.0	Seed and Wang, personal communication	https://dnacore.mgh.harvard.edu/new-cgi-bin/site/pages/viral_genome_sequencing_pages/viral_genome_sequencing_data.jsp
Automated in-house proviral intactness bioinformatic pipeline in Python	Lee et al., 2017	https://github.com/BWH-Lichterfeld-Lab/Intactness-Pipeline
Los Alamos National Laboratory (LANL) HIV Sequence Database Hypermut 2.0	Rose and Korber, 2000	https://www.hiv.lanl.gov/content/sequence/HYPERMUT/background.html
ProSeq-IT	Shao et al., 2020	https://psd.cancer.gov/tools/pvs_annot.php
MUSCLE	Edgar, 2004	http://www.drive5.com/muscle/
Geneious Prime 2021.0.3	Biomatters	https://www.geneious.com/download/
bwa-mem	Li and Durbin, 2009	http://maq.sourceforge.net/
RepeatMasker	Institute for Systems Biology	http://www.repeatmasker.org/
RSEM (v1.2.22)	Li and Dewey, 2011	http://deweylab.github.io/RSEM/
STAR aligner software (2.5.1b)	ENCODE	https://www.encodeproject.org/software/star/
Prism	Graphpad, https://www.graphpad.com/scientific-software/prism	version 8.2.1
R	R Core Team and R Foundation for Statistical Computing, https://www.r-project.org	version 3.5.3
FastQC	Babraham Bioinformatics, https://www.bioinformatics.babraham.ac.uk	version 0.11.9
Samtools	Genome Research Limited, http://www.htslib.org	version 1.14
MACS2	https://github.com/macs3-project/MACS	version 2.1.1.20160309
Recombinant Identification Program	Los Alamos National Laboratory, https://www.hiv.lanl.gov/content/sequence/RIP/RIP.html	
Bowtie2	http://bowtie-bio.sourceforge.net/bowtie2/index.shtml	version 2.2.9
Homer	http://homer.ucsd.edu/homer/interactions/	version 4.10.3
FitHiC2	https://bioconductor.org/packages/release/bioc/html/FitHiC.html	version 1.20.0
FIREcaller	https://github.com/yycunc/FIREcaller	version 1.40
Python	Python Software Foundation, https://www.python.org/	version 3.9
Scikit-learn	https://scikit-learn.org/	Version 0.24.0
Biorender	https://biorender.com	

**Other**		

QX200 Droplet Digital PCR System	Bio-Rad	https://www.bio-rad.com/en-us/life-science/digital-pcr/qx200-droplet-digital-pcr-system
C1000 Touch Thermal Cycler with 96-Well Fast Reaction Module	Bio-Rad	Cat#1851196
Quantify One and ChemiDoc MP Image Lab	Bio-Rad	https://www.bio-rad.com/en-us/product/chemidoc-mp-imaging-system
ThermoMixer C	Eppendorf	Cat#5382000023
96-Well PCR Post Magnet Low Elution Plate	Permagen	Cat#LE400
DynaMag-96 Side Skirted Magnet	ThermoFisher Scientific	Cat#12027
DynaMag-2 Magnet	ThermoFisher Scientific	Cat#12321D
Illumina MiSeq performed by MGH CCIB DNA Core facility	Illumina/MGH CCIB DNA Core	https://dnacore.mgh.harvard.edu/new-cgi-bin/site/pages/index.jsp
FACS Aria Cell Sorter	BD Biosciences	https://www.bdbiosciences.com/en-us/products/instruments/flow-cytometers/research-cell-sorters/bd-facsaria-iii
NextSeq 500 Instrument	Illumina	https://www.illumina.com/systems/sequencing-platforms/nextseq.html

### Resource availability

#### Lead contact

Further information and requests for resources and reagents should be directed to and will be fulfilled by the lead contact, Mathias Lichterfeld (mlichterfeld@partners.org).

#### Materials availability

This study did not generate new unique reagents.

### Experimental model and subject details

#### Study participants

HIV-1-infected study participants were recruited at the Massachusetts General Hospital in Boston, MA. PBMC samples were obtained according to protocols approved by the Institutional Review Board. Clinical characteristics of study participants are summarized in Figure S2. Demographic characteristics of the study patients are as follows:

**Table:** 

Participant	Age at time of study	Sex
P1	54 yrs	M
P2	74 yrs	M
P3	56 yrs	undetermined
P4	65 yrs	M
P5	54 yrs	M
P6	62 yrs	F
P7	60 yrs	M

### Method details

#### HIV-1 DNA quantification by droplet digital PCR

PBMC isolated according to standard protocols were subjected to DNA extraction using commercial kits (Qiagen DNeasy Blood and Tissue Kit, #69504). We amplified total HIV-1 DNA using droplet digital PCR (Bio-Rad), using primers and probes described previously (Lee et al., 2017) (127 bp 5’-LTR-gag amplicon; HXB2 coordinates 684-810). PCR was performed using the following program: 95°C for 10 min, 45 cycles of 94°C for 30s and 60°C for 1 min, 98°C for 10 min. The droplets were subsequently read by a QX200 droplet reader and data were analyzed using QuantaSoft software (Bio-Rad).

#### Separation of genomic DNA and HIV-1 RNA

Cryopreserved PBMCs were thawed, washed, diluted in 96-well plates to single HIV-1-infected cells according to ddPCR results, so that one virally-infected cell was present in approximately 30% of wells, and lysed with Buffer RLT Plus (Qiagen, #1053393). Subsequently, cell lysates were incubated on a thermal mixer (Eppendorf ThermoMixer C, #5382000023) at 1200 rpm for 20 min at room temperature with complexes of magnetic streptavidin beads (Invitrogen Dynabeads MyOne Streptavidin C1, #65002) linked to biotinylated primers targeting defined regions (poly-A, tat-rev, nef, pol, long LTR) of HIV-1 RNA; primer sequences were previously described (Yukl et al., 2018) and modified to permit subsequent amplification with a Smart-seq2 protocol (Picelli et al., 2014). Primer sequences are listed in Table S3. After primer annealing, viral RNA was magnetically separated from genomic DNA according to the G&T-Seq protocol described previously (Bronner and Lorenz, 2019; Macaulay et al., 2016). Briefly, the magnetic beads bound to HIV-1 RNA were immobilized to the bottom of each well with an external magnet and the supernatant containing the genomic DNA fraction was transferred to a new plate. The beads were washed twice in a buffer containing 50 mM Tris-HCl, 7.5 mM KCl, 3 mM MgCl_2_, 10 mM DTT and 0.5% Tween 20; after each wash, the supernatants from each well were collected and added to the corresponding genomic DNA solutions. In a modification of the RNA-binding process for participants 2, 3, 5 and 6, biotinylated poly-A, nef, pol and long LTR primers were added sequentially in 10-minute intervals, rather than simultaneously, since this facilitated the capture and later detection of elongated HIV-1 RNA species. In selected cases, these experiments were performed using an RNA standard (ATCC, VR-3245SD) with known viral copy numbers spiked into a background population of 10,000 cells from an HIV-1-negative person for technical validation of the assay workflow.

#### HIV-1 cDNA synthesis, amplification, and detection

The bead-bound HIV-1 RNA was subjected to reverse transcription with SuperScript II Reverse Transcriptase (Invitrogen, #18064022) using a master mix containing 10 mM dNTPs, 6 mM MgCl_2_, 1 M betaine, 5 mM DTT, 1 U/μL SUPERase⋅In RNase Inhibitor (Invitrogen, #AM2694), 2 μM template-switching oligonucleotide (5’-AAGCAGTGGTATCAACGCAGAGTACATrGrG+G-3’) and 20 U/μL reverse transcriptase in SuperScript II RT First-Strand Buffer. This mix was incubated on a ThermoMixer at 1500 rpm for 60 min at 42 °C, 30 min at 50 °C and 10 min at 60 °C. Subsequently, cDNA was amplified by PCR with KAPA HiFi HotStart ReadyMix (Roche, #7958935001) and 300 nM ISPCR primer (5’-AAGCAGTGGTATCAACGCAGAGT-3’) using a modified Smart-seq2 protocol, as described in the G&T-Seq procedure (Macaulay et al., 2016); for participants 2, 3, 5 and 6, HIV-1-specific primers for poly-A, nef, tat-rev, pol and long LTR viral transcripts (Yukl et al., 2018) were added after the initial 10 Smart-seq2 amplification cycles to complete an additional 25 amplification cycles. Amplified cDNA was subjected to absolute quantification using ddPCR with primers and probes targeting different regions of HIV-1 transcripts, as previously described (Yukl et al., 2018). All primer sequences are listed in Table S3.

#### Whole genome amplification

Isolated gDNA was incubated with AMPure XP magnetic beads (Beckman Coulter, #A63882) and immobilized at the side of each well with an external magnet. After several washes with 80% ethanol, each well was subjected to multiple displacement amplification (MDA) with phi29 polymerase (Qiagen REPLI-g Single Cell Kit, #150345 or REPLI-g Advanced Single Cell Kit, #150365) for 4 hours (Kit #150345) or for 2 hours (Kit #150365), per the manufacturer’s protocol for amplification of genomic DNA from single eukaryotic cells. After this unbiased whole genome amplification (Burtt, 2011), DNA was again incubated with AMPure XP beads and washed and eluted in water. gDNA from each well was split and separately subjected to viral sequencing and integration site analysis, as described below.

#### HIV-1 near full-genome sequencing

DNA resulting from whole-genome amplification reactions was subjected to HIV-1 near full-genome amplification using a near full-length amplicon as well as a nonmultiplexed overlapping 5-amplicon approach, as described before (Einkauf et al., 2019). Additionally, amplification of the HIV-1 core promoter and enhancer region (Shukla et al., 2020) was performed, using one of two nested PCRs: one with primer sequences corresponding to HXB2 coordinates 350-372 and 642-661 (first round) and 367-385 and 626-643 (second round); or another nested PCR with primer sequences corresponding to HXB2 coordinates 24-50 and 936-962 (first round) and 76-100 and 797-818 (second round). All primer sequences are listed in Table S3. These promoter PCRs were performed with the following program: 94 °C for 2 min, 30 cycles of 94 °C for 15 s, 60 °C for 30 s, 68 °C for 1 min. PCR products were visualized by agarose gel electrophoresis (Quantify One and ChemiDoc MP Image Lab, BioRad). All near full-length or 5-amplicon positive PCR products, as well as a large number of proviruses with evident major deletions, were subjected to Illumina MiSeq sequencing at the MGH DNA Core facility. Resulting short reads were *de novo* assembled using Ultracycler v1.0 and aligned to HXB2 to identify large deleterious deletions (<8000bp of the amplicon aligned to HXB2), out-of-frame indels, premature/lethal stop codons, internal inversions, or packaging signal deletions (≥15 bp insertions and/or deletions relative to HXB2), using an automated in-house pipeline written in Python programming language (https://github.com/BWH-Lichterfeld-Lab/Intactness-Pipeline), consistent with prior studies (Hiener et al., 2017; Lee et al., 2017, 2019; Pinzone et al., 2019). Presence/absence of APOBEC-3G/3F-associated hypermutations was determined using the Los Alamos National Laboratory (LANL) HIV Sequence Database Hypermut 2.0 (Rose and Korber, 2000) program; hypermutated proviruses that also contained other structural defects were categorized as hypermutated when binning proviruses according to proviral genomic defects. Viral sequences that lacked all mutations listed above were classified as “genome-intact” sequences. Proviral sequences identified as “genome-intact” with this algorithm were also classified as “genome-intact” using an alternative analysis procedure (Shao et al., 2020). Sequence alignments were performed using MUSCLE (Edgar, 2004) and Geneious Prime 2021.0.3 (geneious.com). Phylogenetic distances between sequences were examined using UPGMA trees in Geneious Prime. Viral sequences were considered clonal if they had identical integration sites and 3 or fewer mismatches between proviral sequences; single nucleotide variations in primer binding sites were not considered for clonality analysis.

#### Integration site analysis

Integration sites associated with each viral sequence were obtained using integration site loop amplification (ISLA), using a protocol previously described (Wagner et al., 2014) and DNA produced by whole-genome amplification as template. Resulting PCR products were subjected to next-generation sequencing using Illumina MiSeq. MiSeq paired-end FASTQ files were demultiplexed; small reads (142 bp) were then aligned simultaneously to human reference genome GRCh38 and HIV-1 reference genome HXB2 using bwa-mem (Li and Durbin, 2009). Biocomputational identification of integration sites was performed according to previously-described procedures (Wagner et al., 2014): Briefly, chimeric reads containing both human and HIV-1 sequences were evaluated for mapping quality based on (i) HIV-1 coordinates mapping to the terminal nucleotides of the viral genome, (ii) absolute counts of chimeric reads, and (iii) depth of sequencing coverage in the host genome adjacent to the viral integration site. The final list of integration sites and corresponding chromosomal annotations was obtained using Ensembl (v86, www.ensembl.org), the UCSC Genome Browser (www.genome.ucsc.edu) and GENCODE (v25, www.gencodegenes.org). Repetitive genomic sequences harboring HIV-1 integration sites were identified using RepeatMasker (www.repeatmasker.org). Cells in which multiple HIV-1 integration sites were detected, either as a result of multiple HIV-1-infected cells being present in one limiting-dilution well or multiple HIV-1 proviruses being integrated into a single cell, were excluded from analysis; the latter possibility is unlikely (Josefsson et al., 2011).

#### HIV-1 reactivation assays

To evaluate HIV-1 RNA expression in response to stimulation, we isolated CD4+ T cells from participant-derived PBMC (Stemcell EasySep™ Human CD4+ T Cell Isolation Kit, #17952), followed by dilution to single HIV-1-infected cells for incubation with 81 nM PMA and 1.34 μM ionomycin (Biolegend Cell Activation Cocktail (without Brefeldin A), #423302) with 20 U/μL recombinant IL-2 and 400 nM AZT; simultaneously, unstimulated autologous cells were plated in the same way for control purposes. After 12 hours of incubation, stimulated and unstimulated CD4+ T cells were subjected to the PRIP-Seq protocol for analysis of HIV-1 integration sites, proviral sequences and HIV-1 RNA expression from individual HIV-1-infected cells.

#### Evaluation of epigenetic and chromosomal contact features

PBMC from three ART-treated HIV-1 participants were used for parallel analysis of CD4 T cells by RNA-Seq, ATAC-Seq, and Hi-C, as described below. ChIP-Seq data were obtained from primary memory CD4 T cells included in the ROADMAP database (Kundaje et al., 2015) or from activated CD4 T cells included in the ENCODE project (ENCODE Project Consortium, 2012). Cytosine methylation data were obtained from the iMethyl database (Komaki et al., 2018).

#### Cell sorting

Briefly, total PBMC were stained with monoclonal antibodies to CD4 (clone RPA-T4, Biolegend, #300518), CD3 (clone OKT3, Biolegend, #317332), CD45RO (clone UCHL1, Biolegend, #304236) and CCR7 (clone G043H7, Biolegend, #353216). Afterwards, cells were washed and CD45RO^+^ CCR7^+^ (central-memory) and CD45RO^+^ CCR7^-^ (effector-memory) and CD3^+^ CD4^+^ (total) CD4^+^ T cells were sorted in a specifically designated biosafety cabinet (Baker Hood), using a FACS Aria cell sorter (BD Biosciences) at 70 pounds per square inch. Cell sorting was performed by the Ragon Institute Imaging Core Facility at MGH and resulted in isolation of lymphocytes with the defined phenotypic characteristics of >95% purity.

#### RNA-Seq

RNA was extracted from total CD4^+^ T-cell populations using a PicoPure RNA Isolation Kit (Applied Biosystems, #0204). RNA-Seq libraries were generated as previously described (Trombetta et al., 2014). Briefly, whole transcriptome amplification and tagmentation-based library preparation was performed using Smart-seq2, followed by sequencing on a NextSeq 500 Instrument (Illumina). The quantification of transcript abundance was conducted using RSEM software (v1.2.22; (Li and Dewey, 2011) supported by STAR aligner software (STAR 2.5.1b) and aligned to the hg38 human genome. Transcripts per million (TPM) values were then normalized among all samples using the upper quantile normalization method. Integration site features were calculated individually using each of the three RNA-Seq datasets from the three assayed participants; medians or averages of the three datasets were used for statistical analyses.

#### ATAC-Seq

A previously-described protocol with some modifications (Corces et al., 2016, 2017) was used. Briefly, 20,000 total CD4 T cells were centrifuged at 1500 rpm for 10 min at 4°C in a precooled fixed-angle centrifuge. All supernatant was removed and a modified transposase mixture (including 25 μl of 2x TD buffer, 1.5 μl of TDE1, 0.5 μl of 1% digitonin, 16.5 μl of PBS, 6.5 μl of nuclease-free water) was added to the cells and incubated in a heat block at 37°C for 30 min. Transposed DNA was purified using a ChIP DNA Clean & Concentrator Kit (Zymo Research, #D5205) and eluted DNA fragments were used to amplify libraries. The libraries were quantified using an Agilent Bioanalyzer 2100 and the Q-Qubit™ dsDNA High Sensitivity Assay Kit (Invitrogen, #Q33230). All Fast-ATAC libraries were sequenced using paired-end, single-index sequencing on a NextSeq 500/550 instrument with v2.5 Kits (75 Cycles). The quality of reads was assessed using FastQC (https://www.bioinformatics.babraham.ac.uk). Low quality DNA end fragments and sequencing adapters were trimmed using Trimmomatic (http://www.usadellab.org). Sequencing reads were then aligned to the human reference genome hg38 using a short-read aligner (Bowtie2, http://bowtie-bio.sourceforge.net/bowtie2/index.shtml) with the nondefault parameters “X2000”, “nonmixed” and “nondiscordant”. Reads from mitochondrial DNA were removed using Samtools (http://www.htslib.org). Peak calls were made using MACS2 with the callpeak command (https://pypi.python.org/pypi/MACS2), with a threshold for peak calling set to FDR-adjusted p<0.05. Integration site features were calculated individually using each of the three ATAC-Seq datasets from the three assayed participants; medians or averages of the three datasets were used for statistical analyses.

#### *Hi*-C

We performed in situ Hi-C on sorted total CD4 T cells, central-memory and effector-memory CD4 T cells as previously described with minor modifications (Díaz et al., 2018). Briefly, one million cells were crosslinked in 1% formaldehyde (Sigma, #F8775-25ML) for 10 min, followed by quenching with glycine (Sigma, #50046-50G). Cell nuclei were permeabilized with 0.4% sodium dodecyl sulphate (SDS) solution and chromatin was digested using 100 U of MboI (NEB, #R0147L). Overhangs generated by the restriction enzyme were filled using Klenow DNA polymerase (NEB, #M0210L) and a mix of dNTPs including biotin-14-dCTP (Invitrogen, #19518018). DNA fragments were then ligated using T4 DNA ligase (NEB, #M0202). After reversion of crosslinking, cellular proteins were digested with Proteinase K (NEB, #P8107), followed by DNA extraction using Phenol-Chloroform-Isoamyl alcohol. The extracted DNA was subjected to digestion of contaminating RNA with RNase A (ThermoFisher, #EN0531). Samples were sheared using a Covaris E220 instrument with a target size of 300-500 base pairs (140W peak incident power, each 67s, 10% duty, 200 cycles/burst) at the MGH NextSeq Core facility. Biotinylated fragments were isolated with Dynabeads MyOne Streptavidin C1 beads (Invitrogen, #65002). DNA linked to the Dynabeads was end-repaired using the End Prep Enzyme Mix and subsequently used for library construction with the NEBNext Ultra DNA Library Prep Kit for Illumina Sequencing (NEB, #E7370S). Final amplification of the libraries was done in 4 parallel reactions per sample according to the following program: 98 °C for 1 min, (98 °C for 10 s, 65 °C for 75 s, ramping 1.5 °C/s) repeated 12–20 times, 65 °C for 5 min, 4 °C. The indexed samples were processed individually and double size-selected using SPRISelect beads (Beckman Coulter, #B23318). Final Hi-C libraries were quantified using the Qubit dsDNA HS assay kit (Invitrogen, #Q33230) and a High Sensitivity D1000 kit (Agilent, #5067-5585) on a Tapestation 4200 (Agilent). The library was then sequenced on an Illumina NextSeq 500/550 (2x80 bp paired-end; NextSeq 500/550 High Output kit v2.5-150 cycles). HomerTools in the software suite HOMER (version 4.10.3) was used to truncate raw sequencing reads at the restriction enzyme cutting site (Heinz et al., 2010), followed by aligning reads to the human reference genome (GRCh38) with Bowtie2 (version 2.3.4.3) (Langmead and Salzberg, 2012). The genome was tiled into bins with defined resolutions using the default method in HOMER to normalize the total read counts in each region. TADs were determined using default utilities from HOMER. Independently, we called significant inter-chromosomal and intrachromosomal interactions using FitHiC2 with Knight-Ruiz matrix balancing (Knight and Ruiz, 2013) and FDR-adjusted p-values < 0.05 as a cutoff (Ay et al., 2014; Kaul et al., 2020). Frequently interacting regions (FIREs) were called using FIREcaller (version 1.10) (Crowley et al., 2021). For data analysis, reads from autologous central-memory, effector-memory and total CD4 T cells were combined to generate a pooled dataset with overrepresentation of reads from memory cells, to account for the fact that most HIV-1-infected cells are included in the memory cell compartment (Chomont et al., 2009). The numbers of total unique sequencing reads in these pooled datasets in the three assayed participants were 1,424,507,007; 1,685,072,487; and 2,400,898,418, respectively. Integration site features were calculated individually using each of the three Hi-C datasets; medians or averages of the three datasets were used for statistical analysis.

### Quantification and statistical analysis

#### Statistics

Data are presented as circos plots, pie charts, bar charts, line graphs and box and whisker plots, showing the median, the 25% and 75% percentiles, and maximum/minimum values. Differences were tested for statistical significance using Mann-Whitney U tests (two-tailed), Fisher’s exact tests (two-tailed) or G-tests, as appropriate. Longitudinal correlations were evaluated using Kendall’s rank correlation coefficient (τ). p-values of <0.05 were considered significant. Analyses were performed using Prism (GraphPad Software, Inc.), Python (Python Software Foundation), and R (R Foundation for Statistical Computing (R Development Core Team, 2019)).

#### Logistic regression model

The Scikit-learn module (Pedregosa et al., 2012) in Python was used to develop a machine learning model to predict the transcriptional activity of HIV-1 integration sites. Briefly, the entire dataset of proviral species was randomly split into a training set with 80% of observations and a test set with 20% of observations, with each set containing the same proportion of observations from each participant and timepoint and the same proportion of RNA+ and RNA- proviruses. A logistic regression model was then trained on the training dataset with HIV-1 Long LTR RNA detection as the target variable. During training, stratified 10-fold cross-validation was used to determine the optimal data standardization and regularization strategies, and ultimately the model with the highest mean cross-validated area under the receiver operating characteristic curve (ROC AUC) was selected for evaluation with the test dataset.

## Data Availability

• This paper does not report original code. • Data were deposited to Gene Expression Omnibus (GEO) with the following accession numbers: RNA-Seq and ATAC-Seq: GSE144334, Hi-C: GSE168337 • Proviral integration sites and their transcriptional activity are listed in Tables S1 and S2**.** • Proviral sequences: Due to study participant confidentiality concerns, viral sequencing data cannot be publicly released but will be made available to investigators upon reasonable request and after signing a coded tissue agreement. • Any additional information required to reanalyze the data reported in this paper is available from the lead contact upon request.

## Supplemental information

Table S1. List of proviral species, their corresponding chromosomal integration features, and their HIV-1 RNA expression profiles in ex vivo assessments of study subjects 1–6, related to Figures 1, 2, 3, 4, 5, and 6

Table S2. List of proviral species, their corresponding chromosomal integration site features, and their HIV-1 RNA expression profiles after in vitro stimulation with PMA/ionomycin or incubation with control media in study subjects 4 and 7, related to Figure 7

Table S3. List of oligonucleotides used in the PRIP-seq assay, related to STAR Methods
