# Supplementary material for: Parallel analysis of transcription, integration, and sequence of single HIV-1 proviruses
Source: Cell. 2022 Jan 20;185(2):266–282.e15. doi: 10.1016/j.cell.2021.12.011 (PMC8809251; doi:10.1016/j.cell.2021.12.011)
Supplement: Table S3. List of oligonucleotides used in the PRIP-seq assay, related to STAR Methods [file mmc3.pdf]

Table S3 (Related to STAR Methods): Oligonucleotide sequences used in PRIP-Seq assay

Workflow	Amplicon/ Target (HXB2 Coordinates)	Round/ Orientation	Oligo Name	Oligo Sequence (5'->3')
Proviral DNA Sequencing	Promoter (76-818)	1F	24F	CGAAGACAAGATATCCTTGATCTGTGG
		1R	962R	CTACAGCCTTCTGATGTTTCTAACAGG
		2F	76F	CTGATTAGCAGAACTACACACCAGG
		2R	818R	CCGCTTAATACTGACGCTCTCG
	Promoter (367-643)	1F	350F	GGGACTTTCCACTGGGGACTTTC
		1R	661R	GCTTTCAGGTCCCTGTTCGG
		2F	367F	ACTTTCCAGGGAGGCGTGG
		2R	Kumar R	GGGCGCCACTGCTAGAGA
	A1mod2 (638-2724)	1F	U5-623F	AAATCTCTAGCAGTGGCGCCCGAACAG
		1R	NE1	CCACTAACTTCTGTATGTCATTGACAGTCCAGCT
		2F	U5-638F	GCGCCCGAACAGGGACYTGAAARCGAAAG
		2R	ProC-	GAGTATTGTATGGATTTTCAGGCCCAAT
	Pol (2011-3798)	1F	5CP1	GAAGGGCACACAGCCAGAAATTGCAGGG
		1R	RT3.1	GCTCCTACTATGGGTTCTTTCTCTAACTGG
		2F	2.5	CCTAGGAAAAAGGGCTGTTGGAAATGTGG
		2R	RT3798R	CAAACTCCCACTCAGGAATCCA
	C (3626-5980)	1F	RT3597mixF	AAAACAGGAAARTATGCAA
		1R	SC05R	AGCTCTTCGTCGCTGTCTCCGCTT
		2F	RT3626F	TGCCCACACTAATGATGTAA
		2R	SC02R	CTTCCTGCCATAGGAGATGCCTA
	A2 (5550-7760)	1F	VP5450F	CAGGACATAACAAGGTAGGATC
		1R	CO602	GCCCATAGTGCTTCCTGCTGCTCCCAAGAACC
		2F	VP5549F	AGAGGATAGATGGAACAAGCCCCAG
		2R	V3CR	TGCTCTTTTTTCTCTCTSCACCACT
	B2 (7652-9610)	1F	GP41Fo	TTCAGACCTGGAGGAGGAGATAT
		1R	3LTRi	TCAAGGCAAGCTTTATTGAGGCTTAA
		2F	GP41Fi	GGACAATTGGAGAAGTGAATTAT
		2R	3UTRi	AGGCTTAAGCAGTGGGTTCCCTAG
Reverse Transcription	Long LTR (643)	RT	Bio Long LTR	/5BiotinTEG/AAGCAGTGGTATCAACGCAGAGTACGGGC GCCACTGCTAGAGA
	Pol (2662)	RT	Bio Pol	/5BiotinTEG/AAGCAGTGGTATCAACGCAGAGTACCAAAT TTCTACTAATGCTTTTATTTTTTC
	Nef (9040)	RT	Bio Nef	/5BiotinTEG/AAGCAGTGGTATCAACGCAGAGTACTGTAA GTCATTGGTCTTAAAGGTACCTGAGG
	PolyA (9635+25)	RT	Bio PolyA	/5BiotinTEG/AAGCAGTGGTATCAACGCAGAGTACTTTTT TTTTTTTTTTTTTTTTTTTTTTGAAG
	Tat-Rev (8459)	RT	Bio Tat-Rev	/5BiotinTEG/AAGCAGTGGTATCAACGCAGAGTACGGATC TGTCTCTGTCTCTCTCTCCACC
	Read-through (582; 9667)	RT	Bio Readth	/5BiotinTEG/AAGCAGTGGTATCAACGCAGAGTACAGAGT CACACAACAGACGG
cDNA Amplification	NA	NA	Template-switching oligo (TSO)	AAGCAGTGGTATCAACGCAGAGTACATrGrG+G-3
	NA	NA	ISPCR	AAGCAGTGGTATCAACGCAGAGT
cDNA ddPCR	Long LTR (522-643)	F	Kumar F	GCCTCAATAAAGCTTGCCTTGA
		R	Kumar R	GGGCGCCACTGCTAGAGA
		R (alternate)	625R	TTTTCCACACTGACTAAAAKGGTC
		Probe	Kumar P	/56-FAM/CCAGAGTCA/ZEN/CACAACAGACGGGCACA/ 3IABkFQ/
	Pol (2536-2662)	F	Pol mf299	GCACTTTAAATTTTCCCATTAGTCCTA
		R	Pol mf1	CAAATTTCTACTAATGCTTTTATTTTTTC
		Probe	Pol P	/56-FAM/AAGCCAGGA/ZEN/ATGGATGGCC/3IABkFQ/
	Nef (8883-9040)	F	F8883-03	GGTGGGAGCAGYATCTCGAGA
		R	R9040-10	TGTAAGTCATTGGTCTTAAAGGTACCTGAGG
		Probe	P8967-50	/56-FAM/CCAGGCACA/ZEN/AKCAGCATT/3IABkFQ/
	PolyA (9496-9635+25)	F	Freadth-2	GCCCTCAGATGCTRCATATAA
		R	5T25	TTTTTTTTTTTTTTTTTTTTTTTTTTGAAG
		Probe	Preadth-1	/56-FAM/TGCCTGTAC/ZEN/TGGGTCTCTCTGGTTAG/ 3IABkFQ/
	Tat-Rev (5956-8459)	F	mf1	CTTAGGCATCTCCTATGGCAGGAA

		R	mf83	GGATCTGTCTCTGTCTCTCTCTCCACC
		Probe	Mf226mod	/56-FAM/ACCCGACAG/ZEN/GCC/3IABkFQ/
	Read-through (411-582; 9496-9667)	F	Freadth-2	GCCCTCAGATGCTRCATATAA
		R	Rreadth-1	AGAGTCACACAACAGACGG
		Probe	Preadth-1	/56-FAM/TGCCTGTAC/ZEN/TGGGTCTCTCTGGTTAG/3IABkFQ/
